# Molecular Remodeling of Tip Links Underlies Mechanosensory Regeneration in Auditory Hair Cells

**DOI:** 10.1371/journal.pbio.1001583

**Published:** 2013-06-11

**Authors:** Artur A. Indzhykulian, Ruben Stepanyan, Anastasiia Nelina, Kateri J. Spinelli, Zubair M. Ahmed, Inna A. Belyantseva, Thomas B. Friedman, Peter G. Barr-Gillespie, Gregory I. Frolenkov

**Affiliations:** 1 Department of Physiology, University of Kentucky, Lexington, Kentucky, United States of America; 2 Oregon Hearing Research Center, Oregon Health & Science University, Portland, Oregon, United States of America; 3 Division of Pediatric Ophthalmology, Cincinnati Children's Research Foundation, Cincinnati, Ohio, United States of America; 4 Laboratory of Molecular Genetics, National Institute on Deafness and Other Communication Disorders, National Institutes of Health, Rockville, Maryland, United States of America; The University of Sheffield, United Kingdom

## Abstract

Sound detection by inner ear hair cells requires tip links that interconnect mechanosensory stereocilia and convey force to yet unidentified transduction channels. Current models postulate a static composition of the tip link, with protocadherin 15 (PCDH15) at the lower and cadherin 23 (CDH23) at the upper end of the link. In terminally differentiated mammalian auditory hair cells, tip links are subjected to sound-induced forces throughout an organism's life. Although hair cells can regenerate disrupted tip links and restore hearing, the molecular details of this process are unknown. We developed a novel implementation of backscatter electron scanning microscopy to visualize simultaneously immuno-gold particles and stereocilia links, both of only a few nanometers in diameter. We show that functional, mechanotransduction-mediating tip links have at least two molecular compositions, containing either PCDH15/CDH23 or PCDH15/PCDH15. During regeneration, shorter tip links containing nearly equal amounts of PCDH15 at both ends appear first. Whole-cell patch-clamp recordings demonstrate that these transient PCDH15/PCDH15 links mediate mechanotransduction currents of normal amplitude but abnormal Ca^2+^-dependent decay (adaptation). The mature PCDH15/CDH23 tip link composition is re-established later, concomitant with complete recovery of adaptation. Thus, our findings provide a molecular mechanism for regeneration and maintenance of mechanosensory function in postmitotic auditory hair cells and could help identify elusive components of the mechanotransduction machinery.

## Abstract 2

Backscatter scanning electron microscopy and conventional whole cell patch-clamp experiments reveal a two-step mechanism for the regeneration of tip links, the crucial element of mechanotransduction machinery in the hair cells of the inner ear.

## Author Summary

The inner ear detects sound when stereocilia, the mechanosensory projections on the apical surface of the hair cells, are deflected and tug on tiny extracellular tip links. These links interconnect stereocilia and convey forces to the mechanosensitive transduction channels. Current models postulate a static composition of the tip link with protocadherin 15 (PCDH15) at the link's bottom end and cadherin 23 (CDH23) at the upper end. Tip links are subjected to substantial sound-induced forces. Although hair cells can renew (regenerate) disrupted tip links and restore hearing, the molecular details of this process are unknown. Our study provides mechanistic insight into tip link regeneration. We used backscatter scanning electron microscopy to monitor the distribution of immuno-gold labeled molecular components of the tip links during their re-formation and a conventional whole-cell patch-clamp technique to follow the concomitant recovery of mechano-electrical transduction. According to our data, the mechanotransduction machinery is initially re-established by the formation of functional (mechanotransduction-mediating) links of a previously unknown composition, PCDH15–PCDH15. Transition to the PCDH15–CDH23 composition underlies final maturation of mechanotransduction. This two-step mechanism of tip link regeneration was unexpected. As tip links are continuously stressed by loud sounds and regenerated throughout an organism's life, we provide a plausible molecular mechanism for the life-long maintenance of mechanosensory function in nonregenerating cochlear hair cells.

## Introduction

**Figure 1:**
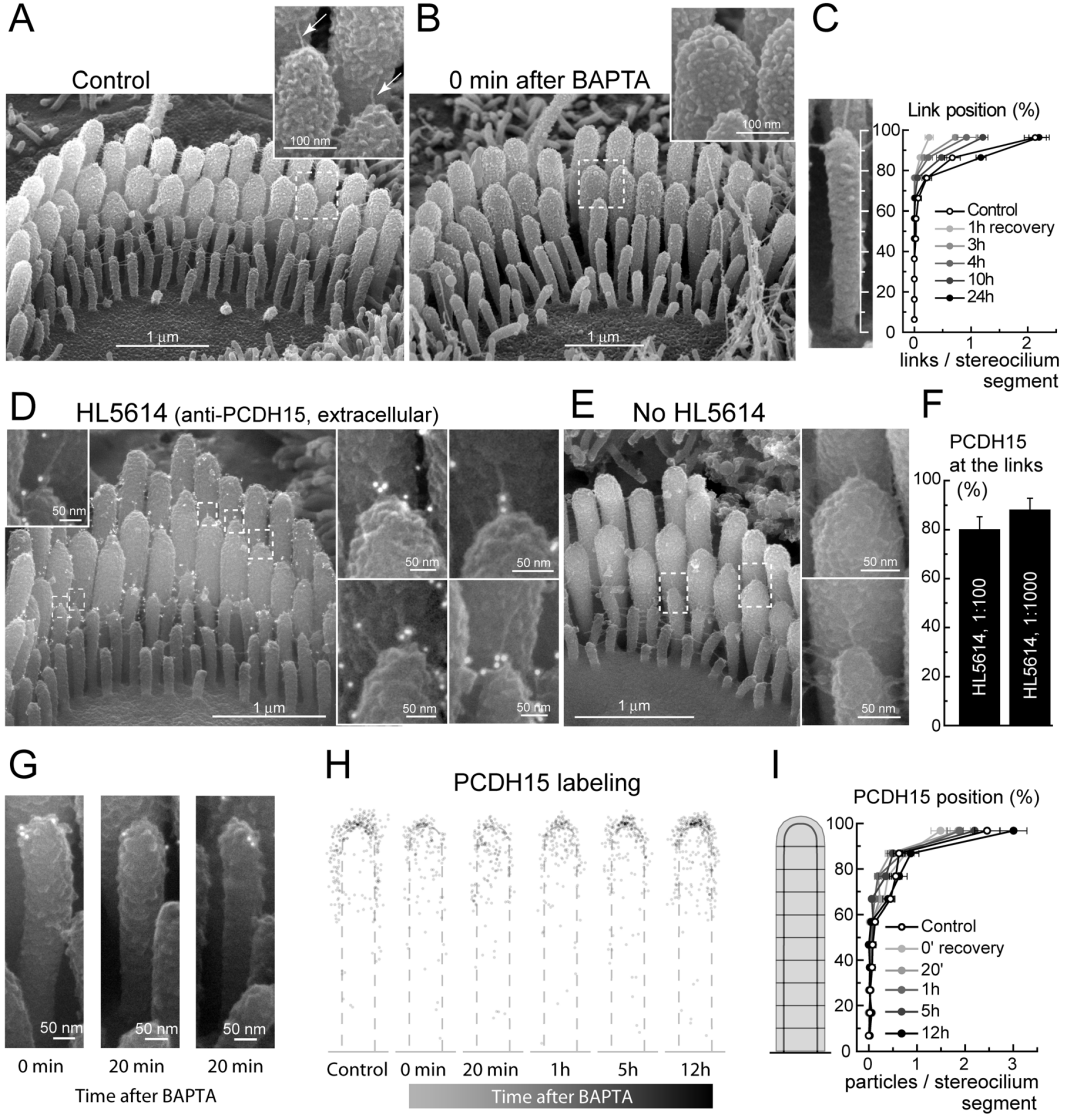
Regenerating stereocilia links appear at the tips but not at the bottom of stereocilia. (A–B) Conventional (secondary electron) SEM images of IHC stereocilia before (A) and immediately after (B) link disruption. Dashed rectangles indicate the areas magnified in insets. Arrows point to the tip links. (C) Distribution of the links along the height of stereocilia (0%, bottom; 100%, top) in the third (shortest) row at different stages of link recovery. (D) Backscatter SEM image of IHC bundle immuno-labeled with anti-PDCH15 antibody, HL5614 (10 nm gold particles seen as white dots). (E) The same as in (D), but primary antibody was omitted. (F) Percentage of immuno-gold particles observed on links of second and third row stereocilia in IHCs at two different dilutions of HL5614. (G) Representative images of HL5614 labeling in third row stereocilia immediately and 20 min after BAPTA treatment. (H) Cumulative distribution of PCDH15 immuno-gold particles on third row stereocilium in control and during link recovery. For each time point, 50–70 stereocilia images were scaled to a common template (dashed line) and the location of every gold particle was shown by a semitransparent grey circle. (I) Distribution of PCDH15 immuno-gold particles along the height of third row stereocilia. Data in panels (C), (F), and (I) are shown as mean ± SE. Age of the cells: P3–4 plus 2–3 days *in vitro* (P3–4+2–3 div).

Mechanosensory stereocilia bundles are arranged in rows of increasing height on the apical surface of the inner ear hair cells (Figure 1A). In mammals, auditory hair cells do not regenerate and therefore stereocilia bundles endure sound-induced deflections throughout an organism's life. When deflected by sound waves, stereocilia slide relative to each other [1] and tug on tiny extracellular tip link filaments [2]. Tip links are oriented obliquely, extending from the tops of shorter row stereocilia to the sides of stereocilia in the neighboring taller row (Figure 1A, inset). Current models of mechano-electrical transduction (MET) postulate that tip link tension controls the opening of the transduction channels that are located at or near the lower ends of the links [3]. Tip links are disrupted *in vivo* by intense acoustical stimulation [4] and can also be ablated *in vitro* by exposure to Ca^2+^-free extracellular medium, which results in the loss of hair cell mechanotransduction [5]. Although mammalian hair cells are terminally differentiated, broken tip links can regenerate and restore mechanosensitivity both *in vitro* [6] and *in vivo* [7]. However, the molecular mechanisms of this regenerative process are unknown, even though it is crucial for maintenance of hearing.

Two protein components of the tip link have been identified, cadherin 23 (CDH23) [8] and protocadherin 15 (PCDH15) [9], which are both required for normal hearing and vision. Mutations of *PCDH15* or *CDH23* cause hearing loss or deaf-blindness [10]–[14]. It was proposed that the tip links are formed by a Ca^2+^-dependent heteromeric interaction between PCDH15 at the lower end of the link and CDH23 at the upper end [15]. This model is further supported by the evidence that tip link regeneration can be inhibited in the presence of soluble extracellular fragments of PCDH15 and CDH23 [16]. However, disrupting the interaction of PCDH15 with CDH23 may have additional effects beyond tip link formation. A point mutation in PCDH15 that inhibits the PCDH15–CDH23 interaction *in vitro* results not only in the loss of tip links but also causes profound changes of the hair bundle structure [17], which were not observed in non-mechanosensitive hair cells of the mice lacking the TMC1/2 proteins, the latest candidates for MET channel subunits [18]. Furthermore, knocking out each of three major classes of PCDH15 alternative C-terminal splice isoforms (CD1, CD2, and CD3) demonstrated that none of them is uniquely required for tip link formation [19]. Therefore, it is still unclear whether or not a functional MET-mediating tip link always consists of a particular heteromeric PCDH15/CDH23 complex or tip links may have a different molecular composition.

To explore the molecular mechanism of tip link regeneration, we developed backscatter electron scanning microscopy to simultaneously visualize nanometer-sized stereocilia links and immuno-gold particles localizing the tip link components PCDH15 and CDH23. We measured the distribution of PCDH15 and CDH23 on the surface of stereocilia and along the length of the tip links during their regeneration. To our surprise, nascent regenerating tip links had PC DH15 at both ends of the link. These newly formed shorter links mediated MET current with a normal amplitude but abnormal Ca^2+^-dependent decay (adaptation). Only later, the heteromeric PCDH15–CDH23 composition of tip links was re-established and the normal adaptation of MET current recovered. Thus, tip link regeneration involves a hitherto unknown remodeling mechanism, in which PCDH15 transiently substitutes for CDH23 to accelerate recovery of hair cell mechanosensitivity.

## Results

**Figure 2:**
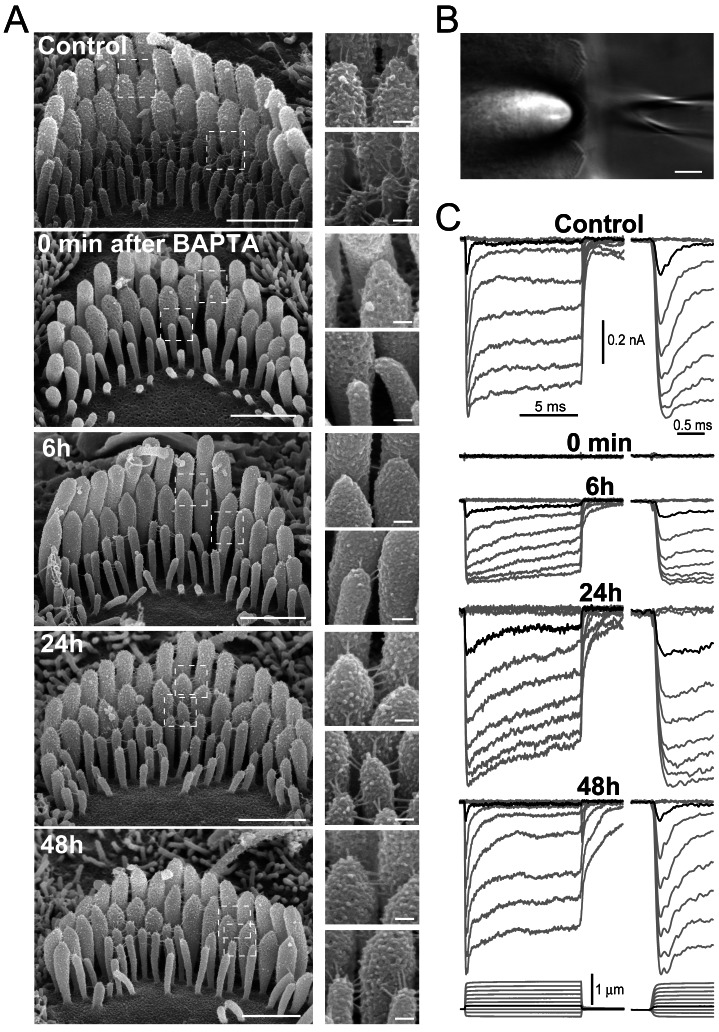
Disruption and subsequent regeneration of stereocilia links and MET current in young postnatal mouse IHCs. (A) Conventional SEM images of IHC stereocilia bundles not treated with BAPTA (control), immediately after BAPTA treatment (0 min), and at 6 h, 24 h, and 48 h of recovery. Insets show magnified images of tip links on the second (top) and third (bottom) row stereocilia indicated by dashed rectangles. Scale bar: 1 µm, main panels; 100 nm, insets. (B) Experimental arrangement with a patch pipette (right) and a piezo-driven probe deflecting stereocilia bundle (left). Scale bar: 5 µm. (C) MET current responses in representative IHCs at the same stages of tip link regeneration as in (A). Traces show MET current evoked by the graded deflections of stereocilia (bottom) with a piezo-driven probe. The beginning of MET current responses is shown on a faster time scale (right). Black traces highlight MET responses to small bundle deflections of 150 nm amplitude. Holding potential was −90 mV. Age of the cells: P3–4+2–4 div; all of them were included in analysis on Figure 4 below.

We studied tip link regeneration in young postnatal (5–7 d old) mouse inner hair cells (IHCs) in cultured organ of Corti explants at an approximately mid-cochlear location. In these IHCs, the developmental changes of the amplitude and Ca^2+^-dependent decay (adaptation) of MET responses are largely complete [20],[21]. Our study capitalizes on the unique sensitivity of stereocilia links in these cells to Ca^2+^ chelation. In young postnatal IHCs, fast exchange to a Ca^2+^-free extracellular solution supplemented with 1,2-bis(o-aminophenoxy)ethane-N,N,N′,N′-tetraacetic acid (BAPTA) disrupts most, if not all, stereocilia links, including tip, “top-to-top,” and “side” links (Figures 1B and 2A). In this study, we defined a link that extends obliquely from the top of a lower row stereocilium to the side of a taller stereocilium in the direction of mechanosensitivity of the bundle as a “tip” link. Any other link originating at the hemisphere of the tip of shorter stereocilium is referred to as “top” link, while the links connecting stereocilia below the tip hemisphere are referred to as “side” links. Note that top and side links in young postnatal IHCs studied here are different from “top connectors” and other links that are insensitive to BAPTA and appear later in development [22]. Stereocilia links in young postnatal IHCs may contain both PCDH15 and CDH23 [9],[23],[24].

Therefore, the complete disappearance of all stereocilia links after BAPTA treatment in young postnatal IHCs allowed us to quantify the re-appearance of links along the full lengths of stereocilia (Figure 1C). This was not possible in previous studies of tip link regeneration in chick basilar papilla [6], bullfrog sacculus [5], or mouse cochlear outer hair cells [16] due to the insensitivity of side links to BAPTA and the very tight spacing between stereocilia in these hair cell types.

### Regenerating Tip Links Assemble Near the Top and Not at the Base of Stereocilia

We measured the distribution of all extracellular links along IHC stereocilia of the third (short) row, because its entire length can be observed by scanning electron microscopy (SEM), often including the space between the third and second row stereocilia. Third row stereocilia are mechanosensitive [3] and have prominent tip links and side links, all of which disappear after BAPTA treatment (Figures 1B and 2A). Newly formed links always localized around the top of a stereocilium during link recovery, including 1 h after BAPTA treatment, when the number of nascent links was very low (Figure 1C). Only a few links were observed early during recovery (15, 30, and 45 min) and they were also located at the tips of stereocilia. We did not observe links in the middle or at the bottom of stereocilia during the first hours of recovery (15 min to 6 h) in any of the 84 organ of Corti explants examined. Rarely were links in these lower positions observed in control untreated samples or in samples that had recovered for 36 h or more. These data suggest that stereocilia links re-assemble at the tops of stereocilia and argue against the hypothesis proposing complete assembly of the MET apparatus, including relevant links, at the base of stereocilium and its subsequent movement to the top as a whole unit [25].

Tip link components may be moved by myosin motors [6] that are able to climb up along the actin core of stereocilia [26]. Therefore, we examined whether the distribution of PCDH15 along stereocilium changes after tip link disruption and during their subsequent regeneration. PCDH15 is expected to be present in third row stereocilia and form the lower ends of nascent tip links [15]. Using an ultra-thin palladium coating, we were able, for the first time to our knowledge, to visualize simultaneously fine extracellular tip links and immuno-gold particles with SEM (Figure 1D). We used a previously validated affinity purified antiserum, HL5614, against an expressed fusion protein corresponding to extracellular sequence from N terminus that is common for numerous splice isoforms of PCDH15 [9]. Both immuno-gold and immunofluorescence HL5614 labeling were not observed when secondary antibodies were used without primary antibodies or in IHC stereocilia of mice homozygous for the *Pcdh15^av-3J^* allele that encodes a truncated PCDH15 lacking the transmembrane and cytoplasmic domains [9], thus validating the specificity of the antibodies (Figures 1E and S1). In control cells untreated with BAPTA, 80%–87% of immuno-gold particles localized to tip links or immature side links (Figure 1F), as expected for the localization of PCDH15 in young postnatal IHCs [9]. One to three gold particles were usually located at the lower end of a tip link (Figure 1D, insets). BAPTA treatment disrupted stereocilia links in wild-type IHCs (Figure 1G) and resulted in re-distribution of PCDH15 around the uppermost surface of the stereocilium (Figure 1H), but did not affect the predominant concentration of PCDH15 labeling toward stereocilia tips (Figure 1I). We concluded that tip links are likely to reassemble using PCDH15 molecules that remain near the tops of stereocilia. Because currently available antibodies against extracellular epitopes of PCDH15 are not isoform-specific, we cannot yet determine which PCDH15 isoforms participate in tip link formation and whether stereocilia tips contain one or more isoforms of PCDH15.

### Regeneration of MET Occurs in Two Distinct Steps

**Figure 3:**
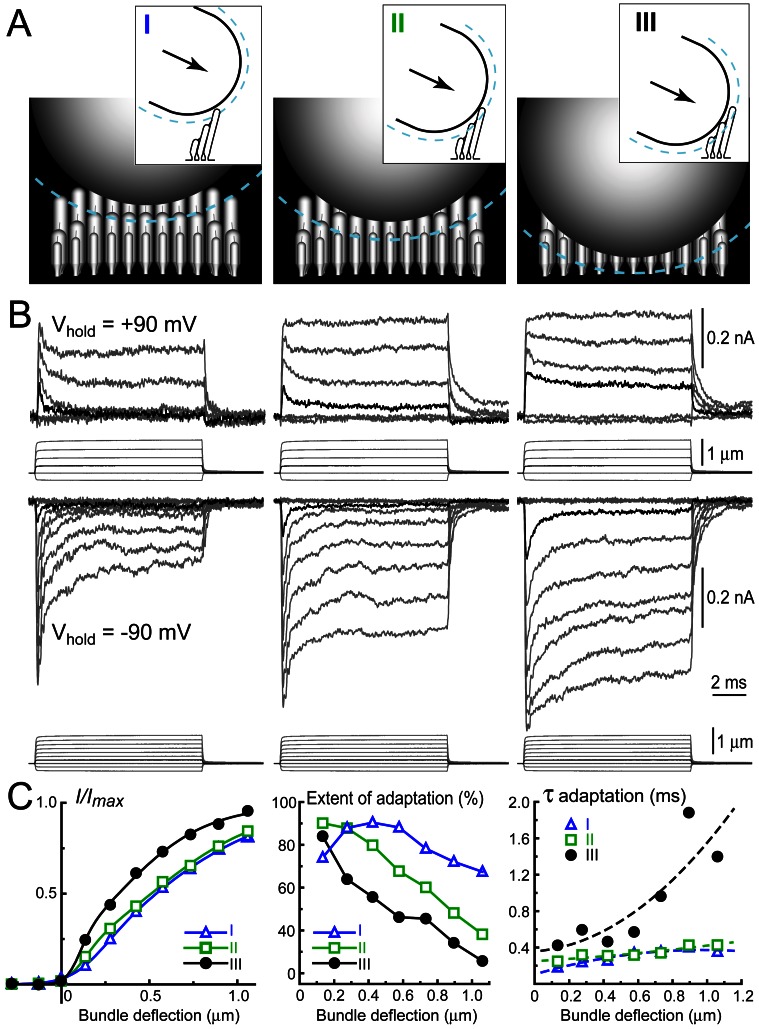
Vertical positioning of the probe is crucial for meaningful MET recordings. (A) Three different probe positions drawn to scale: “very high” (I), “high” (II), and “optimal” (III). Dashed cyan lines indicate the boundary layer of fluid entrained by a fast moving probe. The main panels show a “back view” of the probe deflecting stereocilia bundle, while insets represent side views. In positions (I) and (II), the liquid boundary layer may entrain the outermost stereocilia of the bundle during forward movement of the probe. Therefore, these outermost stereocilia are free to “spring back” when the probe stops. In contrast to bullfrog sacculus stereocilia, which are tightly coupled and move in unison [48], stereocilia of mammalian IHCs are coupled more loosely and are unlikely to move in unison [20],[49]. (B) MET currents (top traces) evoked by the graded deflections of stereocilia (bottom traces) in *the same cell* at different probe positions (I, II, III) at positive (+90 mV) and negative (−90 mV) holding potentials. Positive holding potential prevents Ca^2+^ influx into the cell and, therefore, eliminates Ca^2+^-dependent adaptation. Black traces highlight MET current evoked by the smallest deflection of the probe. (C) The current–displacement relationships of MET current (left); the extent of adaptation, i.e. percent changes of the MET current at the end of bundle deflection of 10 ms duration (middle); and the time constant of MET adaptation, τ_ad_, determined by a single exponential fit (right) at different probe positions (I, blue; II, green; III, black). Data in panel (C) are calculated from traces in panel (B) at holding potential of −90 mV. Age of the cell: P3+4 div.

To examine the properties of MET responses mediated by regenerating tip links, we used conventional whole-cell patch-clamp recording of MET currents evoked by deflection of IHC stereocilia with a piezo-driven rigid probe (Figure 2B). In control cells, hair bundle deflection activates a large inward current with a subsequent prominent decay within a few hundreds of microseconds after activation (Figure 2C, right traces). This fast inhibition of MET channel activity (adaptation) has been described in mammalian IHCs [27] and, despite a multitude of proposed mechanisms, is generally believed to be initiated by Ca^2+^ influx through the MET channels [28]. In contrast to a previous study of MET regeneration in mammalian outer hair cells [16], we monitored MET current not only at negative but also at positive intracellular potentials that eliminate electrochemical driving force for Ca^2+^ influx into the cell (Figure 3A–B). We adjusted the vertical position of the probe to minimize an artifactual and largely unrecognized decay of MET current that is independent of Ca^2+^ influx through MET channels (Figure 3A–B, right). This phenomenon is not observed upon bundle deflection with a fluid-jet, even with relatively fast stimulation [29]. To the best of our knowledge, our stimulating technique is the first one to allow objective determination of the optimal vertical position of the stimulation probe and more accurate recordings of the Ca^2+^-dependent adaptation and deflection sensitivity of the MET current (Figure 3C).

**Figure 4:**
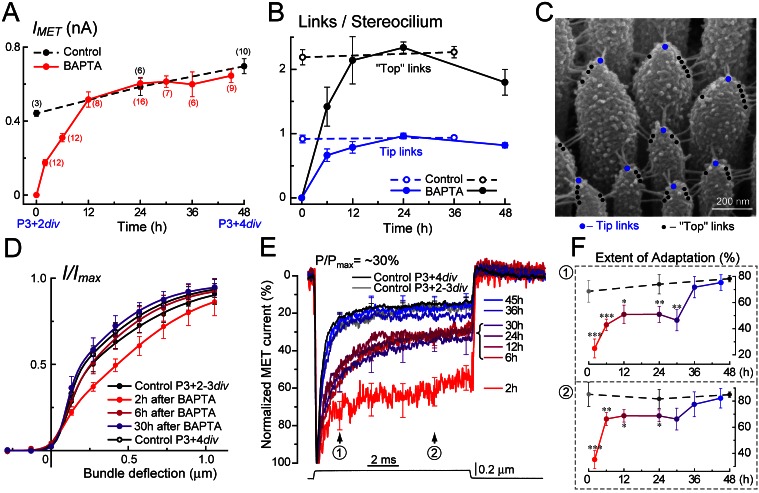
MET regenerates in two distinct steps. (A) Maximal MET current in control IHCs (black) and in IHCs at different times after BAPTA treatment (red). All records were obtained in “optimal” position of the probe (see Figure 3) at holding potential of −90 mV. n, number of cells. (B) Number of “tip” (blue) and “top” (black) links per stereocilium in control IHCs (dashed lines) and in IHCs recovering from BAPTA (solid lines). From 5 to 12 IHCs (154–502 stereocilia) were analyzed for each data point. (C) Representative SEM image of an IHC stereocilia bundle shows tip (blue dots) and top (black dots) links. See the text for exact definitions of “tip” and “top” links. (D) Current–displacement relationships of MET current at different stages of regeneration. Data were fitted to a second-order Boltzmann function and normalized to the maximum MET current obtained from the fit [50]. (E) Overlapping MET currents evoked by a small bundle deflection (150 nm, bottom trace) that increases the open probability of MET channels (P/P_max_) by ∼30%. To compare adaptation at different time points of tip link regeneration, MET responses were normalized to peak current and averaged. Standard errors were calculated for each point of the trace, but shown only at five time points for clarity of the figure. (F) Extent of adaptation calculated at 1.6 ms (1) and 7.6 ms (2) after the beginning of bundle deflection at different stages of MET recovery. Black dashed lines show the data from control IHCs untreated with BAPTA. The same MET records contribute to (A), (D), (E), and (F). All averaged data are shown as mean ± SE. Asterisks indicate statistical significance from the control values: * *p*<0.05; ** *p*<0.01; *** *p*<0.001 (*t* test of independent variables). Age of the cells: P3+2–4 div (dissected at P3 and kept *in vitro* for 2–4 d).

As expected, BAPTA treatment abolished MET current in young postnatal IHCs (Figure 2C). The amplitude of MET current gradually recovered during the 12 h after reintroduction of normal Ca^2+^-containing extracellular medium (Figures 2C and 4A). SEM imaging showed that both tip and top links recovered within the same period (Figure 4B–C). Except for the first 2 h of recovery when the MET current is very small and optimal positioning of the piezo-driven probe cannot be guaranteed, the current–displacement relationship of MET current had the same shape at all stages of MET recovery (Figure 4D). The normal slope of this curve is also consistent with reassembly of mechanosensory links at stereocilia tops rather than their transport from the base of stereocilia, which would result in a less steep current–displacement curve [30].

In spite of a complete restoration of the MET current amplitude by 12 h of recovery (Figure 4A), adaptation did not fully recover, even after 24 h following tip link ablation (Figure 2C; see also [6],[16]). The “fast” component of adaptation dominating at small bundle deflections [31] was affected the most. The time constant of this adaptation component was elevated almost 5-fold in the first hours after BAPTA treatment and returned to control values only after 36 h of recovery (Figure S2A), which is 24 h after complete restoration of the amplitude of MET current and re-establishment of stereocilia links (Figure 4A–B). The “inferred-shift” technique [20],[32] also demonstrated a prominent and long-lasting decrease of the extent of fast adaptation and a small increase of the “slow” component (Figure S2B). Most informative was the alignment of the average normalized MET current at different time points after link ablation (Figure 4E). The aligned traces showed that the extent of adaptation (i.e., percentage of adapting MET current) quickly recovered to an intermediate level but then persisted at this level for more than 24 h, before increasing sharply to normal values sometime between 30 and 36 h after BAPTA treatment (Figure 4F). These data imply a distinct shift in the underlying mechanisms of adaptation during tip link regeneration.

### PCDH15, but Not CDH23, Is Retained at Stereocilia Surface After Tip Link Disruption

**Figure 5:**
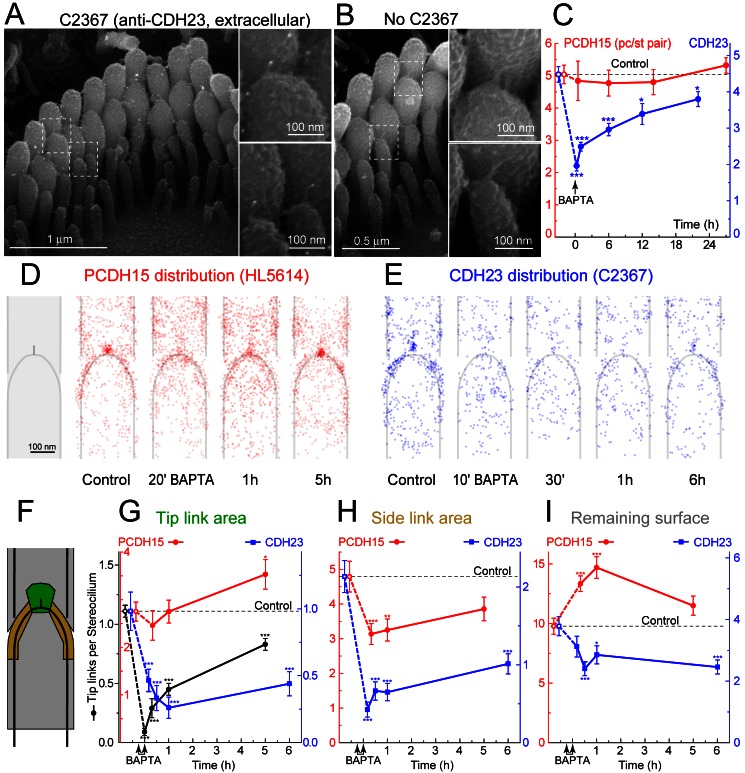
Redistribution of PCDH15 and CDH23 over the surface of stereocilia during tip link regeneration. (A) Backscattered SEM image of an IHC stereocilia bundle, labeled with anti-CDH23 antibody, C2367 (10 nm gold particles seen as white dots). (B) The same as in (A), but primary antibody was omitted. Insets in (A) and (B) show tip links at higher magnification. (C) Average number of all visible anti-PCDH15 (red) and anti-CDH23 (blue) immuno-gold particles on the surface of the first and second row stereocilia (per stereocilia pair) in control IHCs (open symbols) and in IHCs at different time points after BAPTA treatment (solid symbols). (D–E) Distribution of anti-PCDH15 (D, red) and anti-CDH23 (E, blue) immuno-gold particles on the first and second row stereocilia near the tip link region (different stereocilia row than in Figure 1H and different antibody dilution than in Figure 5C). 50 (PCDH15) or 80–90 (CDH23) images of stereocilia pairs were aligned and scaled to a template (leftmost cartoon in D) and the location of each gold particle was marked with a semi-transparent circle. (F) Template showing the regions of interest: tip link area (green), side link area (yellow), and the remaining surface of the stereocilia pair (grey). (G–I) Changes of the number of anti-PCDH15 (red) and anti-CDH23 (blue) immuno-gold particles in the indicated regions of interest. Arrows indicate time of BAPTA treatment. Black circles in (G) show number of tip links per stereocilia pair counted in the immuno-SEM images. The same cells contributed to (D–I). All averaged data are shown as mean ± SE. Asterisks indicate statistical significance from the control values: * *p*<0.05; ** *p*<0.01; *** *p*<0.001 (*t* test of independent variables). Antibody dilutions: C2367, 1∶100; HL5614, 1∶1000 (C), 1∶100 (D–I). Age of the cells: P3+2–3 div.

To elucidate the molecular mechanisms responsible for the two-step MET recovery, we compared the distribution of PCDH15 and CDH23 at the surface of stereocilia before and after BAPTA treatment. We generated antiserum C2367 against the same extracellular epitope of CDH23, PB240 [15], that was used previously for CDH23 localization at the tip link (see Figure S3 for validation). Consistent with previous reports [15],[23],[24], backscatter SEM localized CDH23 to the upper end of the tip links and to the immature side links in young postnatal IHCs (Figure 5A). CDH23 labeling was not observed in CDH23-deficient *Cdh23^v-6J^* mice or in wild-type mice when primary antibodies were omitted (Figures 5B, S3A, and S3C). In general agreement with a previous report [15], BAPTA treatment resulted in redistribution of CDH23 along the length of stereocilia (Figure S4B), but did not result in accumulation of CDH23 at the tip (Figure S4A). The overall number of anti-CDH23 immuno-gold particles in the vicinity of the tip links connecting stereocilia of the first (tallest) and second row decreased significantly after BAPTA treatment and did not fully recover even after 24 h in Ca^2+^-containing medium (Figure 5C, blue). In contrast to CDH23, the amount of anti-PCDH15 immuno-gold particles did not decrease after BAPTA treatment (Figure 5C, red). Although the mechanism of differential loss of CDH23 is unclear, the ectodomain of CDH23 is significantly longer that of PCDH15 [33]. A long unattached ectodomain of CDH23 is more likely to acquire substantial flexibility in low Ca^2+^ environment [34] resulting in “noncanonical” interactions between EC domains of the same or adjacent CDH23 molecules and perhaps the formation of more globular but less stable structures [15],[33].

The loss of CDH23 at the surface of stereocilia after BAPTA treatment was not a species-specific phenomenon. We observed a similar selective decrease of CDH23, but not PCDH15, after BAPTA treatment in chick cochlear hair bundles using polyclonal C2367 and monoclonal G19 [9],[35] antibodies against extracellular epitopes of CDH23 and PCDH15, respectively (Figure S5). We also labeled wild-type mouse IHC stereocilia with a different antibody (TF7) recognizing an intracellular epitope of CDH23 [23] and found a substantial decrease of immunofluorescence after BAPTA treatment (Figure S6), suggesting removal or degradation of stereociliary CDH23.

Examination of the spatial distribution of PCDH15 and CDH23 revealed prominent clustering of immuno-gold particles in the tip link area, which disappeared shortly after stereocilia link ablation (Figure 5D and E). However, PCDH15 and CDH23 responded differently to BAPTA treatment. While anti-CDH23 immuno-gold particles became much less abundant, PCDH15 was merely redistributed along the surface of stereocilia (Figure 5F–I). In the first hour after BAPTA treatment, anti-PCDH15 immuno-gold particles in the tip link region of ∼120×120 nm (Figure 5F, green) were less clustered (Figure 5D), but their total number did not change significantly (Figure 5G). PCDH15 decreased in the side link region (Figure 5H) and increased in nearby areas (Figure 5I), arguing that PCDH15 molecules were free to migrate around to the front of a stereocilium. Later, anti-PCDH15 immuno-gold particles were once again concentrated in the tip link area, where their total number increased relative to control (Figure 5D and G). Although almost 70% of the tip links had reassembled by the 5^th^ hour of recovery (Figure 5G, black), the number of anti-CDH23 immuno-gold particles was still low in all of the analyzed regions of interest and had not yet recovered to control levels (Figure 5G–I, blue). Loss of CDH23 after BAPTA treatment, circumferential migration of PCDH15 shortly after treatment, and its subsequent re-grouping in the tip link area were evident not only in the vicinity of the links connecting first and second row stereocilia (Figure 5) but also in the vicinity of the tip links connecting second and third row stereocilia (data not shown). We therefore hypothesized that PCDH15 compensates for the loss of CDH23 in newly regenerating tip links.

### Molecular Remodeling of the Tip Links During Their Regeneration

**Figure 6:**
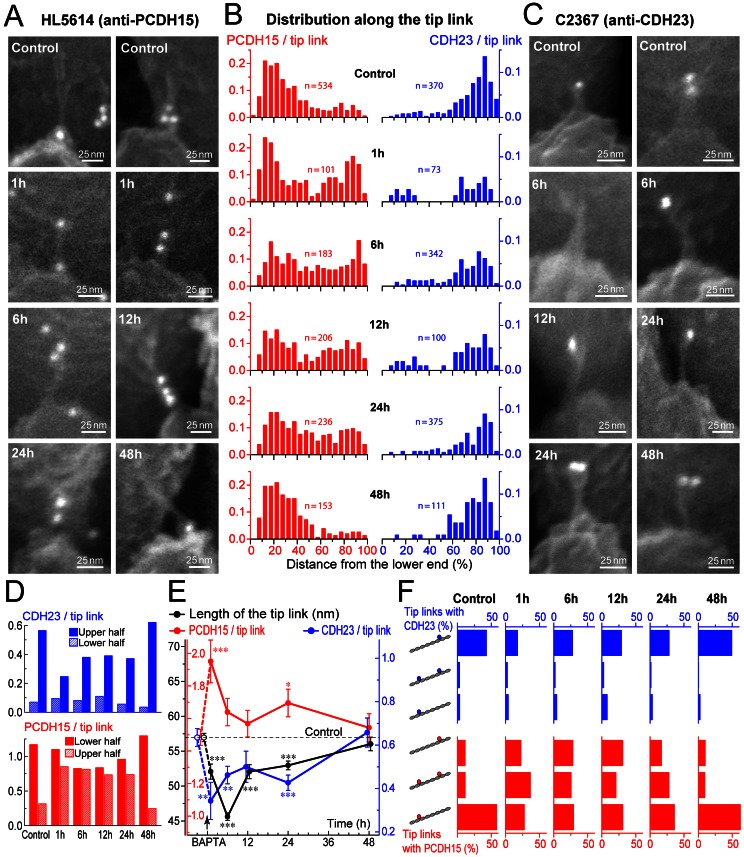
PCDH15 is present equally at both ends of regenerating tip links. (A and C) Representative backscatter SEM images of tip links labeled with HL5614 (A, anti-PCDH15) and C2367 (C, anti-CDH23) antibodies in the IHCs of wild-type mice at different stages of tip link regeneration. (B) Distribution of anti-PCDH15 (red) and anti-CDH23 (blue) immuno-gold particles along the tip link (0%, lower end; 100%, upper end). The number of counts is normalized to the total number of all visible tip links (with and without labeling). Number of analyzed tip links is shown for each histogram. (D) Number of anti-CDH23 (blue bars) and anti-PCDH15 (red bars) immuno-gold particles at lower (left bar at each time point) and upper (right bars) halves of the tip link at different stages of tip link regeneration. (E) Average number of anti-PCDH15 (red) and anti-CDH23 (blue) immuno-gold particles observed on a tip link. Black circles show the average length of the tip links at different stages of regeneration. Note that these values could be compared to the published tip links lengths measured on transmission electron microscopy images only after correction for ∼40% shrinkage due to dehydration, drying, and electron beam irradiation effects and correction for ∼45° viewing angle—i.e., a 150 nm tip link would be observed as a ∼63 nm link by immuno-SEM (150 nm×0.6×Sin(45°) = 63.6 nm). Arrow indicates time of BAPTA treatment. Data are shown as mean ± SE. Asterisks indicate statistical significance from the control values: * *p*<0.05; ** *p*<0.01; *** *p*<0.001. (F) Percentage of tip links having immuno-gold particles only at the upper end of the link (top bars; see also cartoons on the left), both ends of the link (middle bars), and the lower end of the link (bottom bars) among all visible tip links. Data on panels D–F are recalculated from the same set of data that contributed to (B). Age of the cells: P3–4+2–4 div.

Remarkably, the distribution of PCDH15 and CDH23 along the length of the tip link filament changed after BAPTA treatment. We analyzed separately this distribution in the tip links connecting first to second and second to third rows of IHC stereocilia and, because they showed nearly identical results, combined the data (Figure 6). In control untreated IHCs, PCDH15 was localized predominantly to the lower end of the filament (Figure 6A–B), while CDH23 was at the upper end (Figure 6B–C). In nascent regenerating tip links, we observed a large increase of the amount of PCDH15 at the upper ends of the tip links (Figure 6B and D). Throughout regeneration, PCDH15 was distributed almost equally at both ends of the filament (Figure 6B and D). In contrast to PCDH15, a significantly smaller number of anti-CDH23 immuno-gold particles was found on nascent tip links and they were located mostly at the upper end of the filament (Figure 6B and D). The total number of anti-PCDH15 immuno-gold particles per tip link significantly increased in the first hours of tip link regeneration and remained somewhat elevated throughout link regeneration (Figure 6E, red circles), while the number of anti-CDH23 particles per tip link exhibited a reciprocal significant decrease (Figure 6E, blue circles). The apparent length of nascent developing tip links was significantly smaller than the length of tip links in control IHCs, indicating a prolonged period of link remodeling (Figure 6E, black circles). We observed an increased incidence of anti-PCDH15 immuno-gold particles at the upper end or both ends of nascent tip link filaments, as well as multiple particles along the filament's length (Figure 6A). During link regeneration, the percentage of tip links having anti-PCDH15 particles only at the lower end, only at the upper end, and at both ends of the link were approximately equal (Figure 6F), consistent with stochastic labeling of a filament possessing PCDH15 at both ends.

PCDH15 was equally present at both ends of the tip link at 12 and 24 h after BAPTA treatment (Figure 6B and D), when the number of stereocilia links and the amplitude of MET current have recovered completely, but adaptation was still abnormal (Figure 4). Only after ∼48 h of recovery did we observe the heteromeric PCDH15–CDH23 composition of the tip links (Figure 6) and the complete recovery of adaptation (Figure 4E and F).

### Molecular Remodeling of the Tip Links During Postnatal Development

**Figure 7:**
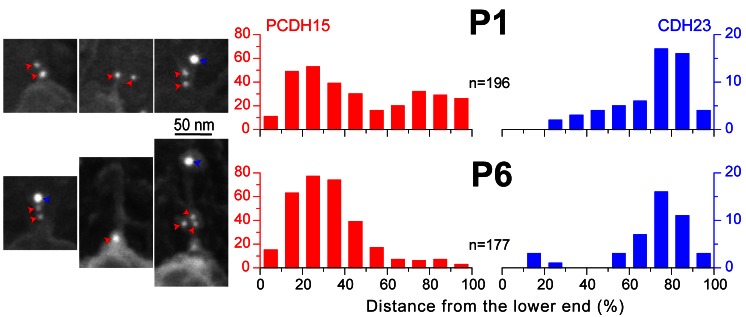
Tip links in mammalian IHCs undergo molecular remodeling during postnatal development. Distribution of anti-PCDH15 (red) and anti-CDH23 (blue) immuno-gold particles along the tip link (0%, lower end; 100%, upper end) in the IHCs of the mouse organ of Corti dissected at P1 (top) and P6 (bottom). For each age, left insets show representative backscatter SEM images of tip links, double labeled with HL5614 (anti-PCDH15) and C2367 (anti-CDH23) antibodies using secondary antibodies conjugated with gold particles of different diameters, 10 and 18 nm correspondingly (indicated by red and blue arrowheads). n, number of analyzed tip links.

Regeneration of tip links and mechanotransduction *in vitro* after BAPTA treatment are thought to recapitulate the developmental assembly of the transduction apparatus *in vivo* [25],[36],[37]. To test whether tip links undergo molecular remodeling during postnatal development, we dissected cochlear epithelia from P1 and P6 mice and double labeled them with HL5614 (anti-PCDH15) and C2367 (anti-CDH23) antibodies, using secondary antibodies conjugated with gold particles of different diameters, 10 and 18 nm correspondingly (Figure 7). Analysis of immuno-gold particle distribution along the length of the tip links in IHCs at mid-cochlear location revealed PCDH15 at both ends of the tip link at P1 and a predominant distribution of PCDH15 at the lower end of the link at P6 (Figure 7, red). CDH23 molecules concentrated mostly at the upper end of the tip link at both ages, P1 and P6 (Figure 7, blue). Thus, the molecular composition of the regenerating tip links after disruption with BAPTA (Figure 6) may indeed recapitulate changes during development (Figure 7).

Postnatal development of MET in mammalian IHCs has not yet been systematically investigated. We previously demonstrated that development of MET current responses and their adaptation are largely complete by P3 in the mouse IHCs in the middle of the cochlea [21]. It is very likely that, similar to the cochlear outer hair cells [30],[37], developing IHCs first acquire the ability to respond to mechanical stimuli and only later exhibit fully mature adaptation. The molecular remodeling of tip links may contribute to this developmental maturation of MET current.

## Discussion

**Figure 8:**
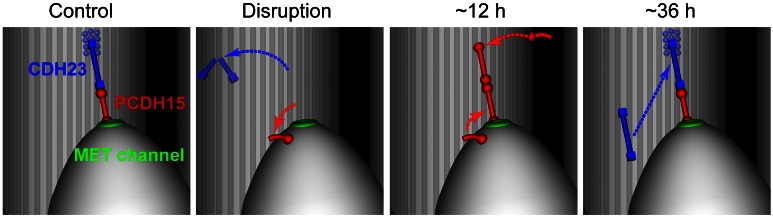
Formation of a transient PCDH15–PCDH15 tip link and its subsequent replacement with PCDH15–CDH23 link. The model assumes that BAPTA disrupts PCDH15–CDH23 bonds [39] and MET channels are bound to or located near the lower end of the tip link [3]. The transduction channel becomes nonfunctional after tip link disruption. Therefore, its location immediately after BAPTA treatment is unknown, although illustrated as present at the tip of stereocilium. Alternatively, MET channels may migrate away from the tip of a stereocilium as a complex with PCDH15 molecules. Harmonin-based complexes linking CDH23 to the cytoskeleton [41] are shown as blue circles near the upper end of the tip link.

Our data revealed a hitherto unknown process of structural remodeling of broken tip links that is necessary for regeneration of MET in auditory hair cells (Figure 8). After stereocilia link disruption, CDH23 largely disappears while PCDH15 migrates from the side link area around the circumference of the stereocilium and becomes available for forming the upper end of a nascent tip link. At the same time, PCDH15 molecules at the lower end of a disrupted tip link also migrate circumferentially but remain at the stereocilia tip. Then, transient tip link filaments are assembled using PCDH15 molecules at both sides of the filament (Figure 8, red). These transient tip links mediate MET current with normal amplitude but an abnormal extent of adaptation. Finally, new or perhaps refolded/repaired CDH23 molecules (Figure 8, blue) replace PCDH15 at the upper end of the tip link, completing the regeneration of mature tip links and the concomitant recovery of MET adaptation. This final step seems to be essential for long-term maintenance of MET because CDH23 is apparently required for MET in mammalian hair cells [38]. The high affinity interaction between CDH23 and PCDH15 [15] may favor the replacement of PCDH15 at the upper end of the tip link with newly synthesized or repaired CDH23, whose appearance in regenerating links is delayed relative to PCDH15.

Because HL5614 antibodies recognize an extracellular epitope that seems to be common to numerous PCDH15 alternative splice isoforms [9] and because these isoforms may have redundant function [19], we cannot distinguish whether nascent regenerating tip links have at their ends identical or different PCDH15 isoforms. It is yet uncertain whether nonclassical cadherins like PCDH15 or CDH23 could form stable extracellular links through homomeric interaction. Evidence for CDH23–CDH23 homomeric interaction have been reported [8], but the same group later suggested that this interaction, along with homomeric PCDH15–PCDH15 interaction, is likely to have a low affinity [15], which is consistent with our data that PCDH15–PCDH15 tip links are only temporary intermediates in the tip link regeneration process. A recent report also provided no direct evidence for homomeric interaction between the first EC domains of either CDH23 or PCDH15 [39]. However, heteromeric interaction of different PCDH15 isoforms is still possible. Alternative splicing of the *Pcdh15* gene can generate several protein products that differ at the N-terminus (Figure S7) and provides a dazzling variety of potential heteromeric interactions between different PCDH15 isoforms, which are known to be targeted specifically to different stereocilia compartments [9]. In addition, a secreted isoform of PCDH15 lacking the transmembrane domain [9],[40] may also be recruited to help span the distance between adjacent stereocilia.

It is yet unclear exactly how molecular remodeling of tip links affects the extent of adaptation of MET responses. It is possible that the decreased length of PCDH15–PCDH15 tip links changes the angle of inclination of tip link and therefore affects the extent of adaptation (Figure S8A–B). However, such changes are predicted to affect the slope of the MET current–displacement curve, which was not observed in our experiments (Figure 4D). The extent of adaptation may also decrease if the elastic harmonin-based complex connecting the upper end of the tip link to the cytoskeleton is replaced to a complex with nonlinear saturating stiffness, for example with PCDH15 that is bound to the cytoskeleton with molecules that “slip down” along the actin core when tip link tension reaches a certain level (Figure S8C). Indeed, recent studies identified harmonin and THMS as interacting partners of CDH23 and PCDH15, correspondingly [41],[42]. These very different interacting partners may contribute to different mechanical properties of macromolecular complexes connecting CDH23 and PCDH15 to the actin cores of stereocilia. Another possibility is that CDH23 at the upper end of the tip link may affect homomeric interaction of parallel PCDH15 filaments at the lower end of the link, thereby affecting the adaptation machinery, or recruit additional adaptation-related components of tip link, which can be seen as a third “foot” at the lower end of the link [43]. In any case, our data open new perspectives to study the molecular basis of MET machinery by characterizing redistribution of the proteins normally associated with PCDH15 and CDH23 during tip link regeneration.

Delayed acquisition of fully developed adaptation is a common finding of studies investigating formation of the mechanotransduction apparatus either after tip link disruption [6],[16] or during development [30],[36],[37]. However, there are different interpretations of these data. According to one model, the fully functional mechanotransduction machinery is assembled somewhere around the base of the hair bundle and transported by myosin-based motors to the tips of stereocilia, where it undergoes final “refinement” [37]. Another model postulates that the mechanotransduction apparatus is assembled at its final location at the tips of the stereocilia from components that arrive there in a stepwise progression [30]. We did not find evidence for upward movement of stereocilia links or PCDH15 molecules along the stereocilium (Figure 1), which is consistent with direct assembly of the transduction apparatus at the tips of stereocilia. On the other hand, replacement of PCDH15 with CDH23 at the final step of molecular remodeling of the tip links may be considered as a “refinement” of the transduction apparatus. We should note, however, one caveat of the direct comparison of our data with the previously published reports. In this study, we investigated regeneration of mechanotransduction in young postnatal mouse IHCs, capitalizing on the unique sensitivity of all stereocilia links in these cells to BAPTA treatment. Previous reports described regeneration or development of mechanotransduction in different hair cell types—e.g., hair cells of chick basilar papilla [6],[36], mouse vestibular hair cells [25], and cochlear outer hair cells of rat [30] and mouse [16],[37]. The diversity of preparations suggests caution in comparing results. For example, an unusually fast recovery of MET current–displacement relationship (Figure 4D) may result from specific mechanical properties of the IHC bundles or from better control of vertical positioning of the stimulating probe in our experiments (Figure 3).

Our data reconcile two previous molecular models of tip link formation. One model proposed that the tip link has a PCDH15–CD3 isoform at the lower end and a PCHD15–CD1 isoform at the upper end of the link [9]. A more recent model suggests that the tip link is built with PCDH15 and CDH23 at the lower and upper ends of the link, correspondingly [15]. We show that the molecular composition of the tip links is not static. During tip link regeneration, the hair cell temporarily utilizes PCDH15 molecules at both ends of the link to recover MET responses until mature PCDH15–CDH23 links are established.

It is generally believed that regeneration of tip links and mechanotransduction *in vitro* recapitulates the assembly of the transduction apparatus *in vivo* (also see Figures 6 and 7) [16],[25],[36],[37]. Therefore, the two-step molecular remodeling process that we have discovered may underlie formation of the tip links in the developing hair bundle, their turnover in the mature bundle, and their regeneration after intense acoustical stimulation in order to safeguard hearing.

## Materials and Methods

### Organ of Corti Explants

Organ of Corti explants were dissected from *C57BL/6* mice at postnatal day 3 or 4 (P3–P4) and cultured on glass bottom Petri dishes (WillCo Wells) for 2–5 d in DMEM medium (Invitrogen) supplemented with 7% fetal bovine serum (Atlanta Biologicals) and 10 mg/L ampicillin (Calbiochem) at 37°C (5% CO_2_). The equivalent age (age of dissection+days *in vitro*) of the organ of Corti explants in this study was P5–P8. Animal procedures were approved by the University of Kentucky Animal Care and Use Committee (Protocol No. 903M2005).

### Stereocilia Link Disruption

The DMEM medium was washed out with Ca^2+^ containing HBSS and then quickly replaced with 1 ml of Ca^2+^-, Mg^2+^-free HBSS (Invitrogen), supplemented with 0.9 mM of MgSO_4_ and 5 mM of BAPTA (Sigma). Following 15 min incubation at room temperature, the BAPTA-containing solution was washed out with normal Ca^2+^-containing HBSS. The samples were then incubated in Ca^2+^-containing DMEM at 37°C as described above for different periods up to 48 h. To confirm tip link disruption, one explant from each set of experiments was washed with Ca^2+^-free HBSS and fixed immediately after BAPTA treatment for SEM observation. Control specimens were incubated with normal Ca^2+^-containing HBSS and then processed identically. The osmolarity of all solutions was adjusted to 320 mOsm with D-glucose. All reported cells were located approximately at the middle of the cochlea.

### Whole-Cell Patch-Clamp Recording

MET responses were recorded using the conventional whole-cell patch-clamp technique as described previously [21],[44]. Experiments were performed at room temperature in Leibovitz (L-15) cell culture medium (Invitrogen) containing the following inorganic salts (in mM): NaCl (137), KCl (5.4), CaCl_2_ (1.26), MgCl_2_ (1.0), Na_2_HPO_4_ (1.0), KH_2_PO_4_ (0.44), MgSO_4_ (0.81).

Hair cells were observed with an inverted microscope (TE2000, Nikon) using a 100× 1.3NA 0.2WD oil-immersion objective and differential interference contrast. To access the basolateral plasma membrane of IHCs, the outermost cells including outer hair cells were removed by gentle suction with a ∼5 µm micropipette. Smaller pipettes for whole-cell patch-clamp recordings were filled with intracellular solution containing (in mM): CsCl (140), MgCl_2_ (2.5), Na_2_ATP (2.5), EGTA (1.0), HEPES (5). The pipette resistance was typically 4–6 MΩ when measured in the bath. Patch clamp recordings were performed with AxoPatch 200B amplifier (Molecular Devices). Series resistance was compensated up to 80% (lag 7–10 µs). After compensation, the time constant of the recording system determined by the “membrane test” feature of the Clampex 9.2 software (Molecular Devices) was in the range of 35–70 µs. IHCs were held at baseline potential of −60 mV between the recordings and at −90 mV or +90 mV for the transient periods of MET recordings. MET recordings at positive (+90 mV) potential were used to adjust the vertical position of the probe along the bundle's height to minimize the artifactual (Ca^2+^-insensitive) component of MET adaptation (Figure 3). MET responses were low-pass filtered at 20 kHz (Bessel type, Stanford Research Systems), acquired at 200 kHz sampling rate, and averaged up to five times.

### Hair Bundle Deflection by a Piezo-Driven Rigid Probe

We deflected hair cell bundles with a stiff glass probe that was fire-polished to match the shape of the hair bundle (∼5–7 µm). The probe was 15–20 mm long, protruding 2–3 mm out of a concentric hole drilled in a plastic screw. The short length of the probe prevents lateral resonances. The probe was moved by a piezo actuator (PA 8/14 SG) custom-modified for a faster response with a time constant of 26–28 µs, driven by a high current amplifier (ENV 800, Piezosystem Jena). Although this actuator is slightly slower than the one used previously [21], its built-in strain gauge sensor provided direct reading of the probe's axial displacement. Command voltage steps were generated by pClamp 9.2 software and low-pass filtered at 25 kHz (Bessel type). The angle between the axis of the probe movement and the bottom surface of a dish was kept at ∼30°. Resting (zero) position of the bundle was estimated by manually advancing the piezo-probe toward the bundle in the excitatory direction until the MET current appeared and then stepping back until the cell current returned to normal. Because this manual technique is not precise, we did not analyze quantitatively the resting open probability of MET channels.

### Conventional SEM

Organ of Corti explants were fixed in 2.5% glutaraldehyde in 0.1 M cacodylate buffer (Electron Microscopy Sciences) supplemented with 2 mM CaCl_2_ for 1–2 h at room temperature. Specimens were dehydrated in a graded series of acetone, critical-point dried from liquid CO_2_, sputter-coated with 4–5 nm of platinum (Q150T, Quorum Technologies, United Kingdom), and observed with a field emission scanning electron microscope (S-4800, Hitachi, Japan).

### Immunofluorescent Labeling

Cultured organ of Corti explants were immersed directly in 4% paraformaldehyde in HBSS. Acutely isolated temporal bones were perfused with this fixative through small holes at the base and the apex of the cochlea. After 1–2 h fixation at room temperature, the organ of Corti was dissected in HBSS. All further steps of immunolabeling were identical for cultured and isolated organ of Corti specimens. For detection of intracellular epitopes with TF7 antibody, samples were permeabilized with Triton X-100 (0.5%) for 10–30 min at room temperature. Nonspecific binding sites were blocked by incubation for 2 h at room temperature with 10% goat or rabbit serum in Ca^2+^, Mg^2+^-free HBSS supplemented with 2 mM EDTA to unmask the epitopes [45]. Samples were incubated overnight with primary antibodies in the blocking solution (1∶100–1∶1000 dilutions for HL5614, 1∶200 for TF7, 1∶100 for C2367). After several rinses in Ca^2+^-free HBSS, samples were incubated for 2 h at room temperature with secondary antibody diluted 1∶200 and supplemented with rhodamine phalloidin (2.5 unit/ml, Invitrogen) to visualize F-actin. We used Alexa Fluor 488 goat anti-rabbit IgG (Invitrogen), DyLight 488-conjugated Affini-Pure Goat Anti-Rabbit IgG (Jackson ImmunoResearch), or Alexa Fluor 488 (Fab′)_2_ fragment of rabbit anti-goat IgG (H+L) (Invitrogen) secondary antibodies. Explants were mounted under cover slips using ProLong antifade kit (Invitrogen) and observed with an Axiovert 200 microscope equipped with a Plan-Apochromat 100× 1.4 NA oil immersion objective and the LSM 5 Live laser confocal scanning module (Carl Zeiss, Germany).

### Chicken Cochlea Immunohistochemistry

Inner ear organs from P0 *Gallus gallus* were dissected in HEPES-buffered HBSS at room temperature. After incubation in 50 µg/mL subtilisin (Sigma) for 20 min, the tectorial membrane was removed using an eyelash and forceps. Organs were incubated in 5 mM BAPTA in Ca^2+^-, Mg^2+^-free HEPES-buffered HBSS for 5 min with agitation by flow from a syringe needle to ensure that tip links were broken. Organs were quickly washed three times in HBSS and immediately fixed in 4% paraformaldehyde for 20 min. Subsequent steps were performed in HBSS containing 5 mM EGTA to unmask extracellular antibody binding sites. Organs were permeabilized in 0.5% Triton X-100 for 5 min and blocked with 3% normal serum, 2% BSA for 2 h. Cochlea were incubated in blocking solution with goat anti-CDH23 antibody C2367 and mouse anti-PCDH15 antibody G19, each at 1∶100, overnight at 4°C. After five washes, organs were again incubated overnight at 4°C with Alexa 488-phalloidin and donkey anti-goat Alexa 546 and donkey anti-mouse Alexa 647 secondary antibodies diluted 1∶300. Following five washes, organs were postfixed in 4% paraformaldehyde for 15 min, mounted in Vectashield, and imaged on an Olympus FV-1000 confocal microscope. Z-stacks were deconvolved using softWoRx Suite (Applied Precision) and analyzed with Imaris 3D reconstruction software (Bitplane).

### Immuno-SEM Labeling

Live cultured organ of Corti explants were observed with an inverted microscope (TE2000, Nikon) and the fibrous material of the tectorial membrane was gently removed with a ∼2–4 µm suction pipette. Explants were then fixed with 4% paraformaldehyde and 0.2% glutaraldehyde in HBSS for 1–2 h at room temperature. Acutely dissected temporal bones were perfused with the same fixative through the small holes at the base and apex of the cochlea. After 1–2 h fixation at room temperature, an organ of Corti was dissected in HBSS. All further steps of immunolabeling were identical for cultured and isolated organ of Corti specimens. Nonspecific binding sites were blocked and the samples were incubated with primary antibody as described above. We used the following 1∶20 diluted secondary antibodies: Immuno-gold Conjugate EM Goat F(ab′)_2_ anti-rabbit IgG:10 nm (BB International) or Immuno-gold Conjugate EM Rabbit anti-goat IgG:10 nm (BB International) for 2 h at room temperature. After secondary antibody application, samples were postfixed in 2.5% glutaraldehyde in 0.1 M cacodylate buffer for 1–2 h at room temperature.

### Immuno-SEM Imaging

Although visualization of immuno-gold particles with a backscatter SEM detector is a previously reported technique, we found that the reported methods are not suitable for imaging stereocilia links. Common carbon coating of samples does allow imaging of immuno-gold particles on stereocilia [45],[46], but a reasonable electric conductivity of the sample is achieved only with a heavy coating of ∼20 nm thickness, which hides stereocilia links. Chromium is another common coating material, but it is susceptible to oxidation, resulting in stereocilia link damage. Using an “ExB filter” of the Hitachi S-4800 instrument, we were able to observe immuno-gold particles on samples coated with a fine 2 nm layer of iridium or in samples prepared with OTOTO [47] technique. However, this approach worked only on relatively flat surfaces. The convoluted surface of the stereocilia bundle generates too many backscatter electrons for a reasonable atomic number (Z-) contrast. After trying carbon, chromium, silver, and iridium coating, we found that a fine coating with palladium allows sufficient Z-contrast and an acceptable resolution of fine surface structures. Therefore, the samples were dehydrated and dried as described above, but sputter coated with 2 nm of palladium. Stereocilia bundles were observed with Hitachi S-4800 field emission scanning electron microscope equipped with YAG detector of backscatter electrons.

### Western Blot

HEK 293T cells were transfected with a mouse Cdh23 expression vector or GFP (negative control) plasmids. Cells were lysed using sonication in the presence of 1% Triton X-100; the extract was centrifuged for 20 min at 16,000 g and 4°C, and the supernatant saved. Proteins in the supernatant were solubilized with SDS sample buffer and separated by SDS-PAGE on a 3%–8% gradient gel. Proteins were transferred to a blotting membrane and antibody C2367 was used at 1 µg/ml to detect CDH23.

## Supporting Information

Figure S1 **Specificity of HL5614 (anti-PCDH15) antibody.** (A–D) Immunofluorescent detection of HL5614 antibodies (green) in the IHC stereocilia counterstained with rhodamine phalloidin (red) in the wild-type (A), control heterozygous (B, *Pcdh15^+/av-3J^*), and homozygous (C, *Pcdh15^av-3J/av-3J^*) *Ames waltzer* mice, as well as in the *Pcdh15^+/av-3J^* mouse when HL5614 primary antibody were omitted (D). (E–F) Backscatter SEM images of immuno-gold labeling of IHC stereocilia bundles with HL5614 antibody in *Pcdh15^+/av-3J^* (E) and *Pcdh15^av-3J/av-3J^* (F) mice. Higher magnification images in the right panels show the areas indicated by dashed rectangles in the left panels. Dilution of HL5614 antibody: (A) 1∶250; (B, C) 1∶1,000; (E, F) 1∶500. All specimens were dissected from littermate mice at postnatal day 8 (P8).
(TIF)

Figure S2 **Delayed recovery of adaptation.** (A) The time constant of adaptation determined by a single exponential fit of the first 2 ms of MET current response evoked by the smallest (150 nm) bundle deflection in the control IHCs (black) and in the IHCs at different time points after BAPTA treatment (red). This time constant reflects mostly “fast” adaption because this component of adaptation dominates in mammalian hair cells at small bundle deflections [31],[44]. (B) Extent of fast (left panel) and slow (right panel) components of adaptation determined by the “inferred-shift” technique [32] in control hair cells (black) and at different stages of hair bundle recovery (red). Asterisks indicate statistical significance of the difference from the control values: * *p*<0.05; ** *p*<0.01; *** *p*<0.001. Age of the cells: P3+2–4 div (dissected at P3 and kept *in vitro* for 2–4 d).
(TIF)

Figure S3 **Specificity of C2367 (anti-CDH23) antibody.** (A) Backscatter SEM images of immuno-gold labeling of IHC stereocilia with C2367 antibody in the control heterozygous *Cdh23^+/v-6J^* (*waltzer*) mouse (top) and homozygous *Cdh23^v-6J/v-6J^* mouse (bottom). *Cdh23^v-6J^* was reported to be a functional null allele due to a nonsense mutation (904G>T; p.Glu302X) encoding CDH23 truncated in the third ectodomain [14]. Right panels show magnified images of the areas indicated by dashed rectangles. Organs of Corti were dissected from littermate mice at postnatal day 7 (P7). (B) Percentage of CDH23 immuno-gold particles in wild-type IHC bundles associated with stereocilia links (top) and average number of particles at the stereocilia of different rows (bottom). Due to predominant localization of CDH23 immuno-gold particles at the upper end of the tip link, the total number of visible CDH23 particles on the stereocilia of the first (tallest) and second rows was larger than that on the stereocilia of the third (shortest) row. Cultured organ of Corti explants (P3+2–3 div) untreated with BAPTA. (C) Immunofluorescence detection of C2367 antibody (green) in the IHC stereocilia counterstained with rhodamine-phalloidin (red) in the control heterozygous *Cdh23^+/v-6J^* mouse (top row), homozygous *Cdh23^v-6J/v-6J^* mouse (middle row), and in the *Cdh23^+/v-6J^* IHCs not treated with C2367 antibody (bottom row). Organs of Corti were dissected from littermate mice at P7. (D) Immunoblot detection of C2367 antigen in HEK293 cells transfected with the full-length mouse cochlea-specific isoform of *Cdh23* (with exon 68) (+) and in control HEK293 cells transfected with GFP (−). Expressed full-length CDH23 migrates at a molecular weight (∼450 kD) that is larger than predicted (∼350 kD), presumably because of posttranslational modifications.
(TIF)

Figure S4 **Redistribution of CDH23 at stereocilia surface after tip link ablation.** (A) Backscatter SEM images of a control untreated IHC (top) and an IHC following 10 min of recovery after BAPTA treatment at 37°C (bottom). Right insets show the tips of stereocilia at higher magnification. (B) Distribution of distances from the tip of a second row stereocilium to the closest CDH23 particle on the adjacent first row stereocilium before link disruption and at different stages of link recovery. This count included only one (closest to the stereocilium tip) particle per each stereocilia pair, irrespective of the presence or absence of a tip link. See Figures 5 and 6 for quantitative assessment of CDH23 changes during link regeneration. Age of the cells: P3+2 div.
(TIF)

Figure S5 **Loss of CDH23 but not PCDH15 immunofluorescence in chick cochlear hair bundles after BAPTA treatment.** (A) Reconstructed orthogonal view of chick cochlear hair bundles stained with anti-CDH23 (C2367) and monoclonal anti-PCDH15 (G19) antibodies in control cells (left panel) and after treatment with 5 mM BAPTA for 5 min (right panel). (B) Average intensity (in arbitrary units) of CDH23 (green) and PCDH15 (blue) immunofluorescence in control bundles (solid bars) and after treatment with BAPTA (hatched bars). ROIs were selected based on actin staining. Mean intensity of CDH23 or PCDH15 fluorescence was measured and normalized to phodamine-phalloidin (F-actin) fluorescence (Control *n* = 120, BAPTA *n* = 95).
(TIF)

Figure S6 **Decrease of intracellular CDH23 from stereocilia bundles after tip link disruption.** Immunofluorescence labeling of IHC bundles with TF7 antibody (green) that recognizes an intracellular epitope of CDH23 [23] at different stages of stereocilia link regeneration. From top to bottom: control sample untreated with BAPTA; sample immediately after BAPTA treatment (0 min); samples after 20 min, 6 h, and 27 h of recovery; another control sample that was processed identically but primary antibodies were omitted. F-actin was counterstained with rhodamine phalloidin (red). Age of the cultured organ of Corti explants: P3+2–3 div.
(TIF)

Figure S7 **Variability at the N-terminus of PCDH15 due to alternative splicing.** Schematic representation of mouse *Pcdh15* alternatively spliced isoforms. In mouse inner ear and retina, at least 24 alternative splice variants are expressed that differ at their N-terminus extracellular cadherin repeats (EC) region, and transmembrane domain [9]. There are also three different cytoplasmic domains (CD1, CD2, or CD3) [9]. Only variants at the amino terminus are shown since one or more of these isoforms are predicted to interact with the extracellular N-terminus domain of CDH23 and PCDH15 itself. (A) Full-length isoform of PCDH15 includes a signal peptide (red), 11 ECs, a single transmembrane domain, and one of the three cytoplasmic domains (accession no. AAG53891). (B) PCDH15 isoform without residues encoded by cassette exon 3 (green) (DQ354396). (C) PCDH15 isoform lacking sequence encoded by cassette exons 3 and 4 (purple) (DQ354402). (D) PCDH15 isoform lacking cassette exons 16 and 17 results in a merge of parts of EC4 and EC5 domains (DQ354400). (E) PCDH15 isoform without the EC2 domain (DQ354401). (F) EC4 cadherin repeat in this isoform has an insertion of seven additional amino acid (DQ354405) encoded by cassette exon 12a. (G) Exons encoding the amino terminus first three EC domains are absent in the mRNA encoding this isoform (DQ354407).
(TIF)

Figure S8 **Potential mechanisms of the dependence of MET adaptation on tip link composition.** (A) Transduction channel (green) in series with a nonstretchable PCDH15–CDH23 tip link [43],[51], “tension release” adaptation element [52], and intracellular elastic elements at lower (*K_1_*) and upper (*K_2_*) ends of the tip link. According to Hooke's law, when the system is stretched by Δx with an external stimulus, MET channel encounters force: *F_peak_* = *K_1_*×*K_2_*/(*K_1_*+*K_2_*)×Δx. Activation of the tension-release element decreases overall elongation of elastic components by Δd and changes the force experienced by the channel: *F_adapted_* = *K_1_*×*K_2_*/(*K_1_*+*K_2_*)×(Δx−Δd) = *F_peak_*−*K_1_*×*K_2_*/(*K_1_*+*K_2_*)×Δd. Assuming the MET channel is an ideal force sensor, extent of adaptation is: Ex = (*F_peak_*−*F_adapted_*)/*F_peak_* = Δd/Δx. At first approximation, Δx is determined by shear displacement between stereocilia multiplied by Sin(Θ), where Θ is the tip link inclination angle [53]. Therefore, the extent of adaptation depends on the geometry of the MET apparatus. (B, left) If a shorter PCDH15–PCDH15 tip link is formed without changes to the stereocilia separation at the level of a tip link, Θ would decrease resulting in a smaller Δx and a larger extent of adaptation. (B, right) If stereocilia are closer to one another during link regeneration because of an increased number of shorter side links, the angle Θ may increase, decreasing the extent of adaptation. It is not possible to detect changes in Θ based on immuno-SEM images. (C) Replacement of CDH23 with PCDH15 at the upper end of the tip link may introduce to the MET apparatus an element with nonlinear stiffness (*K_2_*, red). For example, it may occur if myosin motors connecting PCDH15 to the actin core of stereocilium would not withstand tension above a certain threshold and “slide down” along the actin core. Nonlinear force–displacement relationship of the system (red graph on the right panel) would result in a decreased extent of adaptation, similar to the one observed in our experiments during tip link regeneration (Figure 4E–F).
(TIF)

## Abbreviations

BAPTA: 1,2-bis(o-aminophenoxy)ethane-N,N,N′,N′-tetraacetic acid
div: days *in vitro*
HBSS: Hank's Balanced Salt Solution
IHCs: inner hair cells
MET: mechano-electrical transduction
SEM: scanning electron microscopy
PCDH15: protocadherin 15
CDH23: cadherin 23
