# Supplementary figures and images for: Molecular Remodeling of Tip Links Underlies Mechanosensory Regeneration in Auditory Hair Cells
Source: PLoS Biol. 2013 Jun 11;11(6):e1001583. doi: 10.1371/journal.pbio.1001583 (PMC3679001; doi:10.1371/journal.pbio.1001583)

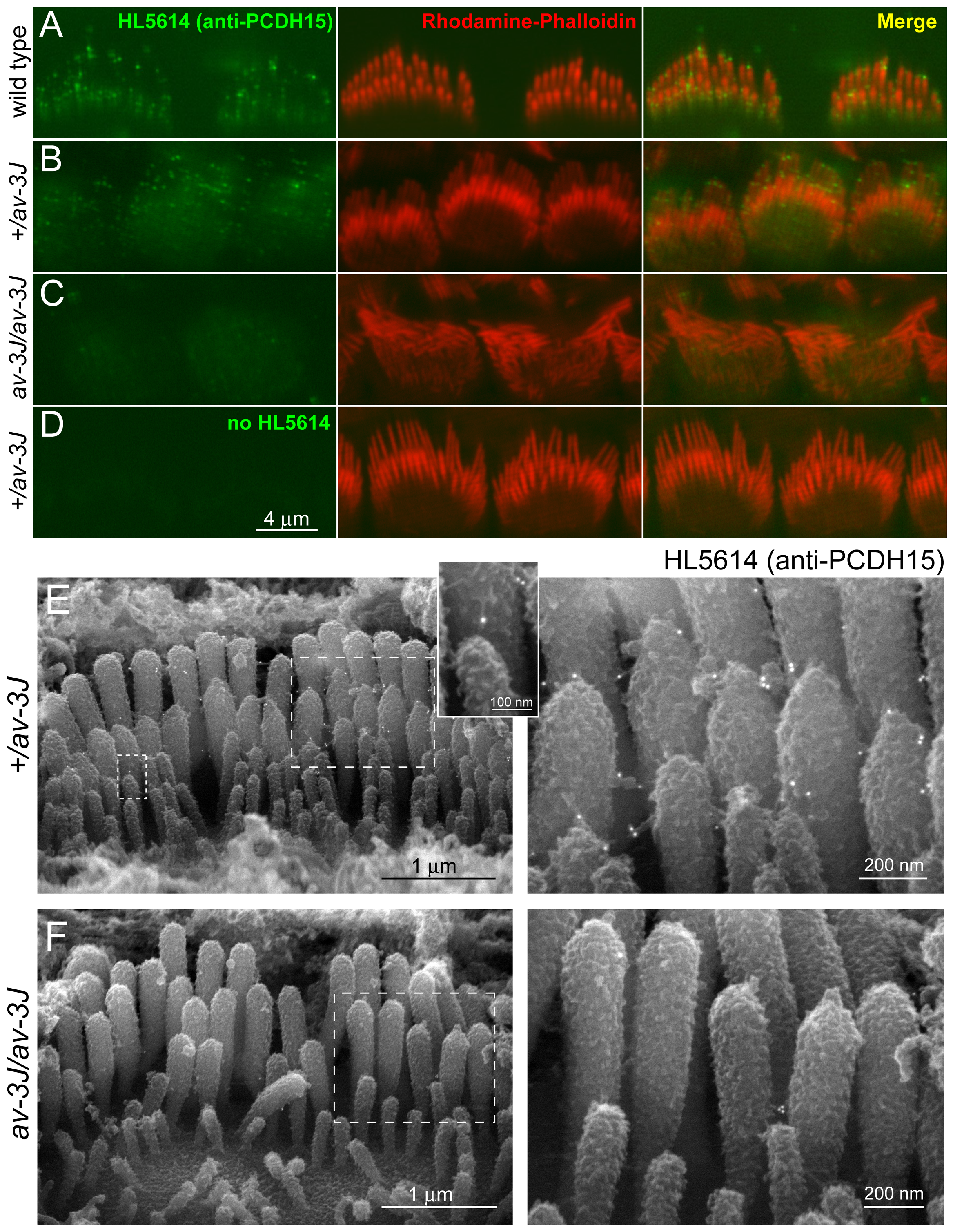

Supplement: Figure S1 — Specificity of HL5614 (anti-PCDH15) antibody. (A–D) Immunofluorescent detection of HL5614 antibodies (green) in the IHC stereocilia counterstained with rhodamine phalloidin (red) in the wild-type (A), control heterozygous (B, Pcdh15+/av-3J), and homozygous (C, Pcdh15av-3J/av-3J) Ames waltzer mice, as well as in the Pcdh15+/av-3J mouse when HL5614 primary antibody were omitted (D). (E–F) Backscatter SEM images of immuno-gold labeling of IHC stereocilia bundles with HL5614 antibody in Pcdh15+/av-3J (E) and Pcdh15av-3J/av-3J (F) mice. Higher magnification images in the right panels show the areas indicated by dashed rectangles in the left panels. Dilution of HL5614 antibody: (A) 1∶250; (B, C) 1∶1,000; (E, F) 1∶500. All specimens were dissected from littermate mice at postnatal day 8 (P8). (TIF) [file pbio.1001583.s001.tif]

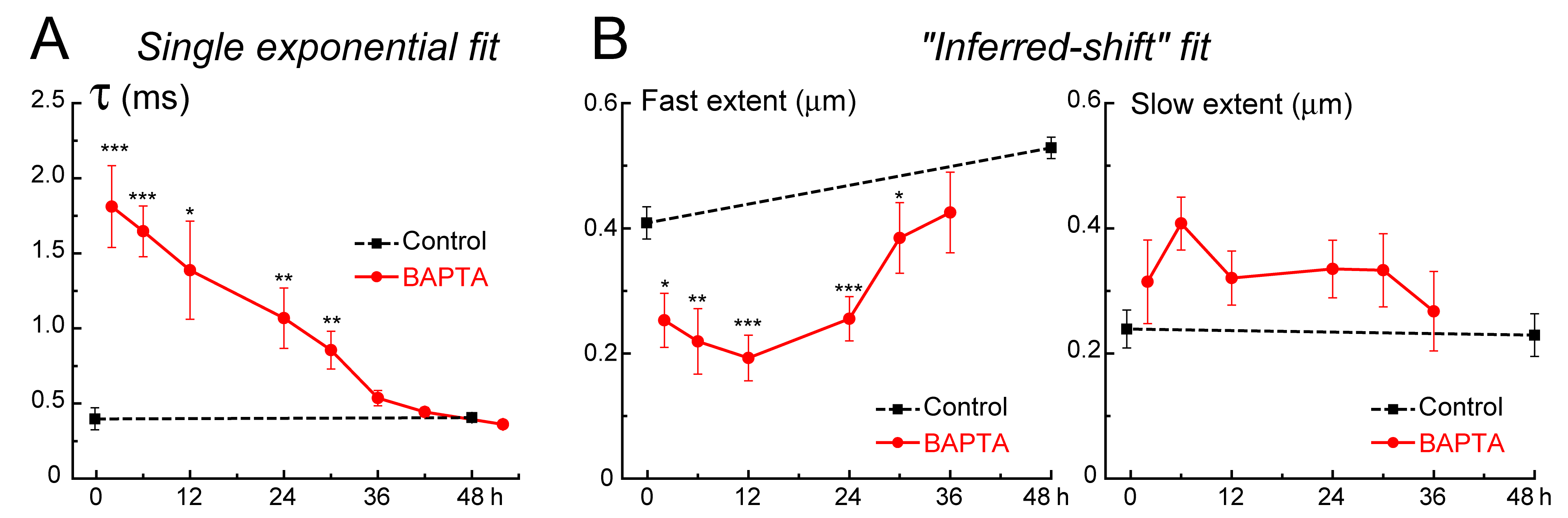

Supplement: Figure S2 — Delayed recovery of adaptation. (A) The time constant of adaptation determined by a single exponential fit of the first 2 ms of MET current response evoked by the smallest (150 nm) bundle deflection in the control IHCs (black) and in the IHCs at different time points after BAPTA treatment (red). This time constant reflects mostly “fast” adaption because this component of adaptation dominates in mammalian hair cells at small bundle deflections [31],[44]. (B) Extent of fast (left panel) and slow (right panel) components of adaptation determined by the “inferred-shift” technique [32] in control hair cells (black) and at different stages of hair bundle recovery (red). Asterisks indicate statistical significance of the difference from the control values: * p<0.05; ** p<0.01; *** p<0.001. Age of the cells: P3+2–4 div (dissected at P3 and kept in vitro for 2–4 d). (TIF) [file pbio.1001583.s002.tif]

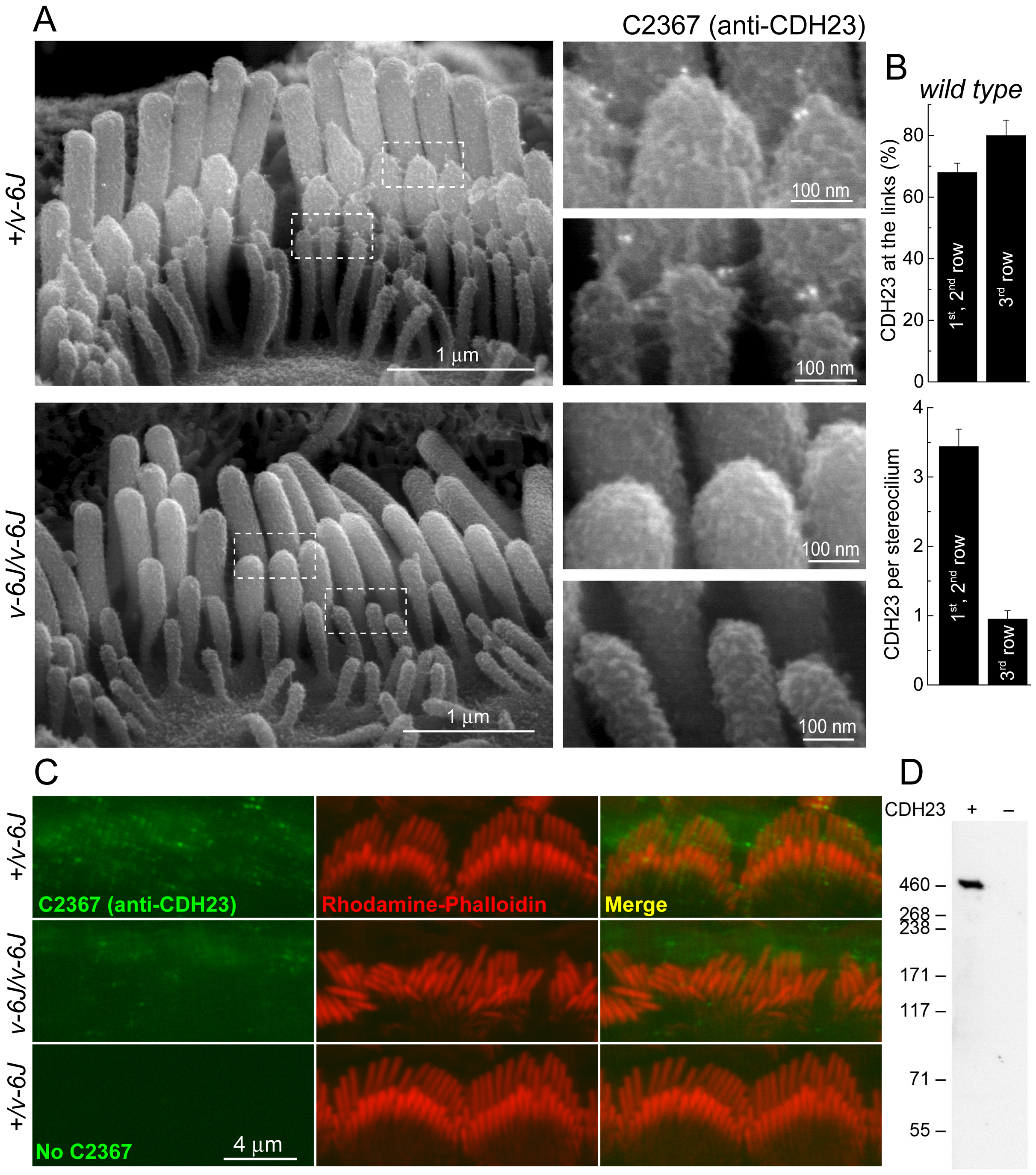

Supplement: Figure S3 — Specificity of C2367 (anti-CDH23) antibody. (A) Backscatter SEM images of immuno-gold labeling of IHC stereocilia with C2367 antibody in the control heterozygous Cdh23+/v-6J (waltzer) mouse (top) and homozygous Cdh23v-6J/v-6J mouse (bottom). Cdh23v-6J was reported to be a functional null allele due to a nonsense mutation (904G>T; p.Glu302X) encoding CDH23 truncated in the third ectodomain [14]. Right panels show magnified images of the areas indicated by dashed rectangles. Organs of Corti were dissected from littermate mice at postnatal day 7 (P7). (B) Percentage of CDH23 immuno-gold particles in wild-type IHC bundles associated with stereocilia links (top) and average number of particles at the stereocilia of different rows (bottom). Due to predominant localization of CDH23 immuno-gold particles at the upper end of the tip link, the total number of visible CDH23 particles on the stereocilia of the first (tallest) and second rows was larger than that on the stereocilia of the third (shortest) row. Cultured organ of Corti explants (P3+2–3 div) untreated with BAPTA. (C) Immunofluorescence detection of C2367 antibody (green) in the IHC stereocilia counterstained with rhodamine-phalloidin (red) in the control heterozygous Cdh23+/v-6J mouse (top row), homozygous Cdh23v-6J/v-6J mouse (middle row), and in the Cdh23+/v-6J IHCs not treated with C2367 antibody (bottom row). Organs of Corti were dissected from littermate mice at P7. (D) Immunoblot detection of C2367 antigen in HEK293 cells transfected with the full-length mouse cochlea-specific isoform of Cdh23 (with exon 68) (+) and in control HEK293 cells transfected with GFP (−). Expressed full-length CDH23 migrates at a molecular weight (∼450 kD) that is larger than predicted (∼350 kD), presumably because of posttranslational modifications. (TIF) [file pbio.1001583.s003.tif]

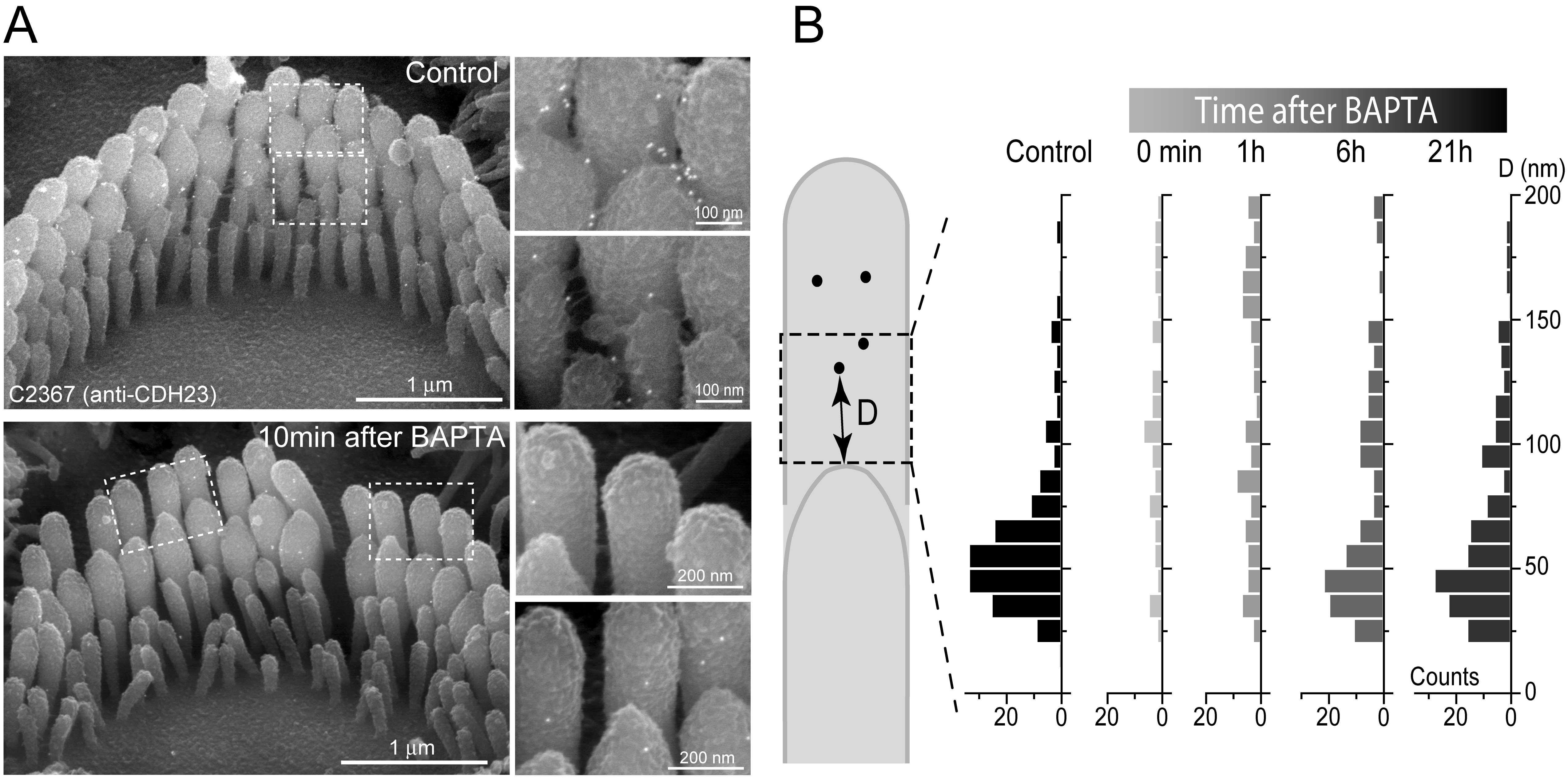

Supplement: Figure S4 — Redistribution of CDH23 at stereocilia surface after tip link ablation. (A) Backscatter SEM images of a control untreated IHC (top) and an IHC following 10 min of recovery after BAPTA treatment at 37°C (bottom). Right insets show the tips of stereocilia at higher magnification. (B) Distribution of distances from the tip of a second row stereocilium to the closest CDH23 particle on the adjacent first row stereocilium before link disruption and at different stages of link recovery. This count included only one (closest to the stereocilium tip) particle per each stereocilia pair, irrespective of the presence or absence of a tip link. See Figures 5 and 6 for quantitative assessment of CDH23 changes during link regeneration. Age of the cells: P3+2 div. (TIF) [file pbio.1001583.s004.tif]

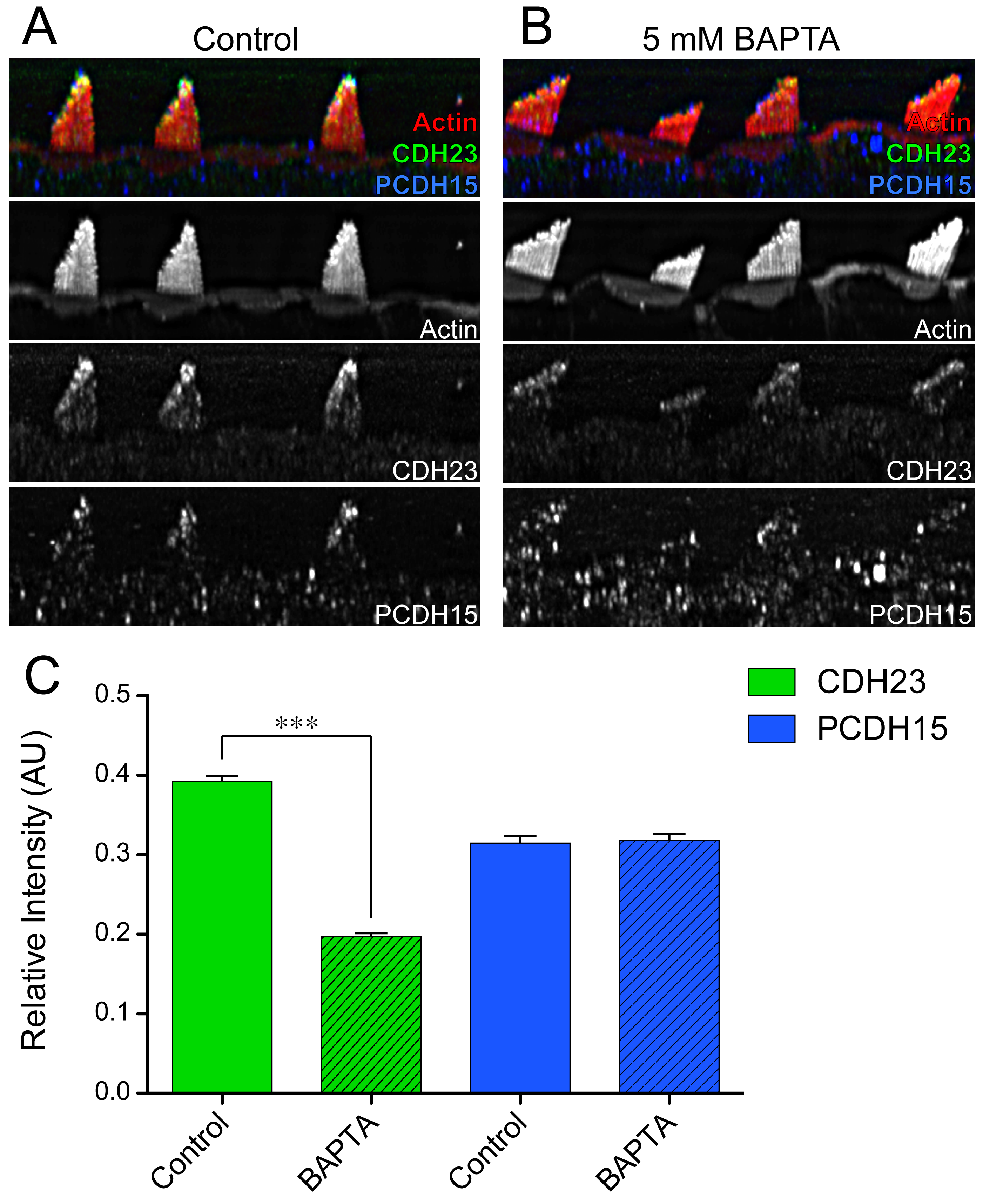

Supplement: Figure S5 — Loss of CDH23 but not PCDH15 immunofluorescence in chick cochlear hair bundles after BAPTA treatment. (A) Reconstructed orthogonal view of chick cochlear hair bundles stained with anti-CDH23 (C2367) and monoclonal anti-PCDH15 (G19) antibodies in control cells (left panel) and after treatment with 5 mM BAPTA for 5 min (right panel). (B) Average intensity (in arbitrary units) of CDH23 (green) and PCDH15 (blue) immunofluorescence in control bundles (solid bars) and after treatment with BAPTA (hatched bars). ROIs were selected based on actin staining. Mean intensity of CDH23 or PCDH15 fluorescence was measured and normalized to phodamine-phalloidin (F-actin) fluorescence (Control n = 120, BAPTA n = 95). (TIF) [file pbio.1001583.s005.tif]

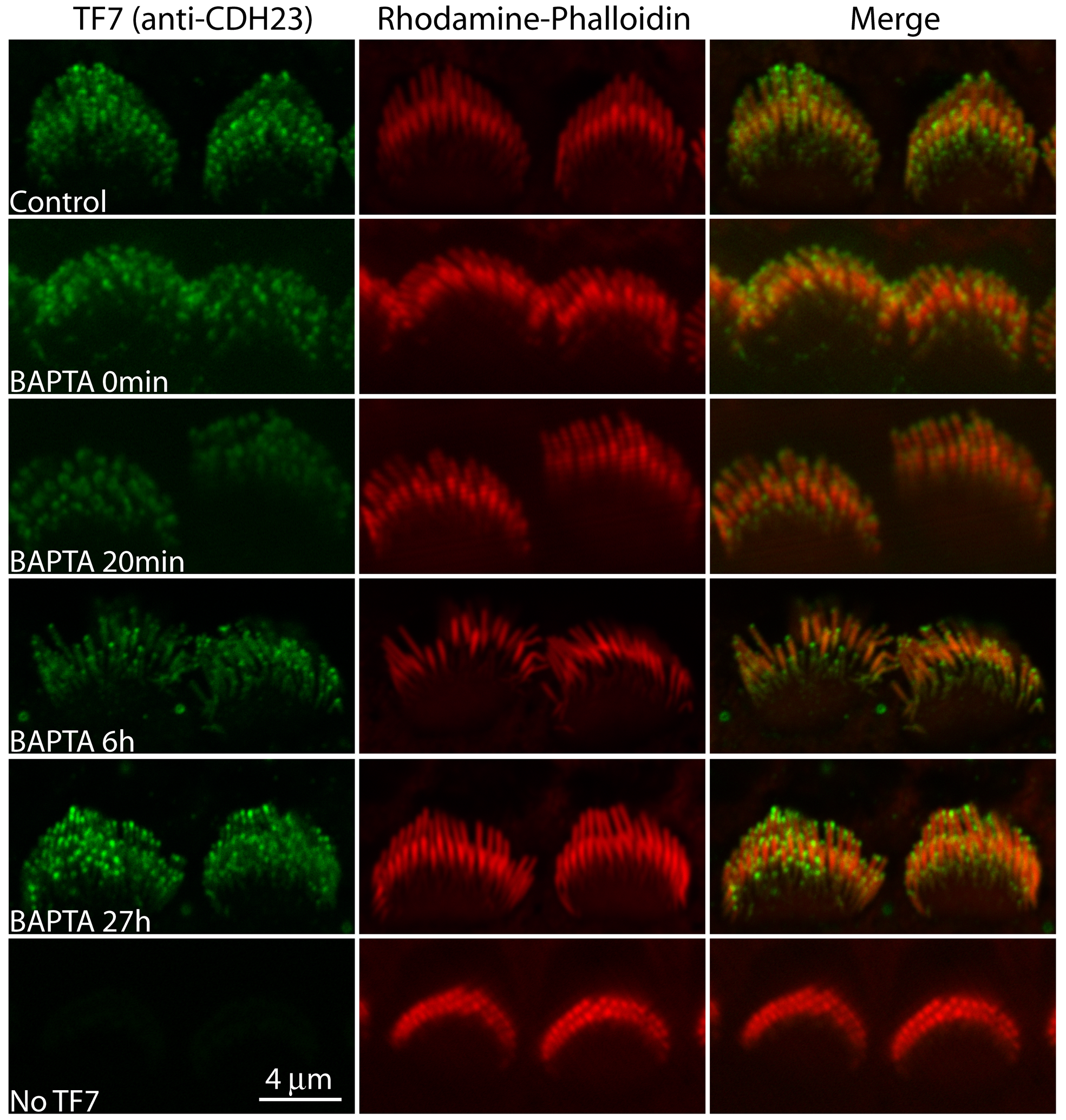

Supplement: Figure S6 — Decrease of intracellular CDH23 from stereocilia bundles after tip link disruption. Immunofluorescence labeling of IHC bundles with TF7 antibody (green) that recognizes an intracellular epitope of CDH23 [23] at different stages of stereocilia link regeneration. From top to bottom: control sample untreated with BAPTA; sample immediately after BAPTA treatment (0 min); samples after 20 min, 6 h, and 27 h of recovery; another control sample that was processed identically but primary antibodies were omitted. F-actin was counterstained with rhodamine phalloidin (red). Age of the cultured organ of Corti explants: P3+2–3 div. (TIF) [file pbio.1001583.s006.tif]

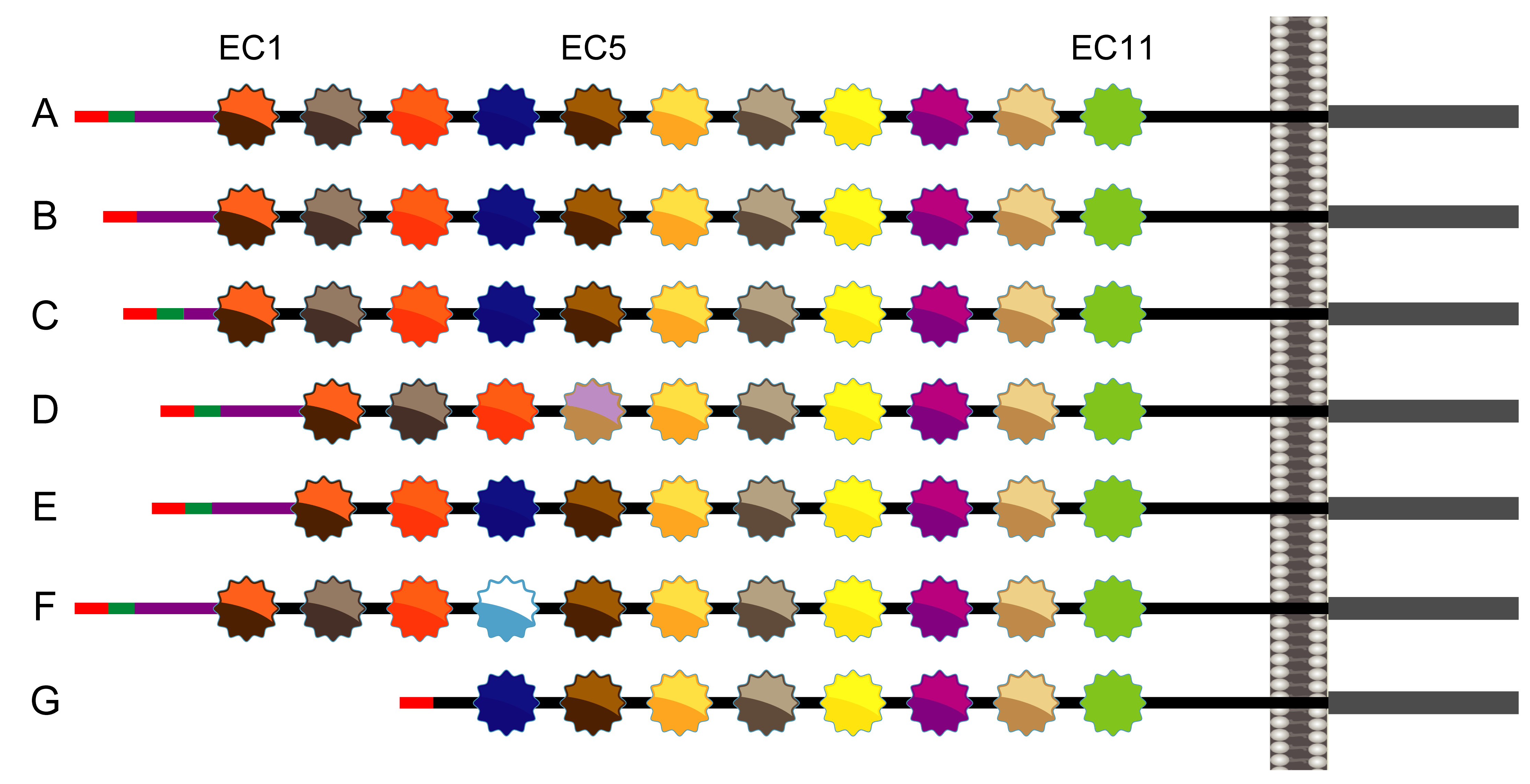

Supplement: Figure S7 — Variability at the N-terminus of PCDH15 due to alternative splicing. Schematic representation of mouse Pcdh15 alternatively spliced isoforms. In mouse inner ear and retina, at least 24 alternative splice variants are expressed that differ at their N-terminus extracellular cadherin repeats (EC) region, and transmembrane domain [9]. There are also three different cytoplasmic domains (CD1, CD2, or CD3) [9]. Only variants at the amino terminus are shown since one or more of these isoforms are predicted to interact with the extracellular N-terminus domain of CDH23 and PCDH15 itself. (A) Full-length isoform of PCDH15 includes a signal peptide (red), 11 ECs, a single transmembrane domain, and one of the three cytoplasmic domains (accession no. AAG53891). (B) PCDH15 isoform without residues encoded by cassette exon 3 (green) (DQ354396). (C) PCDH15 isoform lacking sequence encoded by cassette exons 3 and 4 (purple) (DQ354402). (D) PCDH15 isoform lacking cassette exons 16 and 17 results in a merge of parts of EC4 and EC5 domains (DQ354400). (E) PCDH15 isoform without the EC2 domain (DQ354401). (F) EC4 cadherin repeat in this isoform has an insertion of seven additional amino acid (DQ354405) encoded by cassette exon 12a. (G) Exons encoding the amino terminus first three EC domains are absent in the mRNA encoding this isoform (DQ354407). (TIF) [file pbio.1001583.s007.tif]

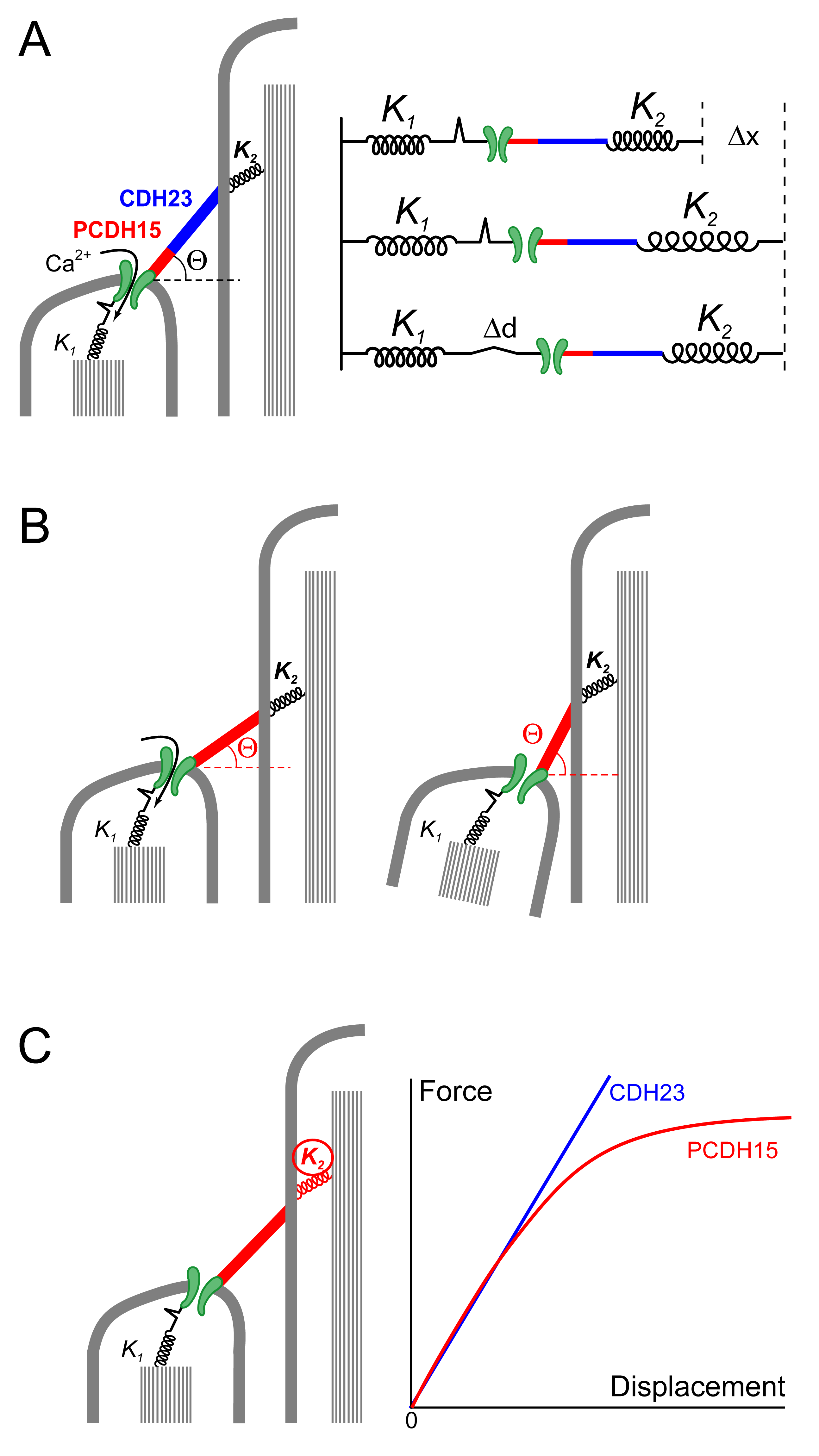

Supplement: Figure S8 — Potential mechanisms of the dependence of MET adaptation on tip link composition. (A) Transduction channel (green) in series with a nonstretchable PCDH15–CDH23 tip link [43],[51], “tension release” adaptation element [52], and intracellular elastic elements at lower (K1) and upper (K2) ends of the tip link. According to Hooke's law, when the system is stretched by Δx with an external stimulus, MET channel encounters force: Fpeak = K1×K2/(K1+K2)×Δx. Activation of the tension-release element decreases overall elongation of elastic components by Δd and changes the force experienced by the channel: Fadapted = K1×K2/(K1+K2)×(Δx−Δd) = Fpeak−K1×K2/(K1+K2)×Δd. Assuming the MET channel is an ideal force sensor, extent of adaptation is: Ex = (Fpeak−Fadapted)/Fpeak = Δd/Δx. At first approximation, Δx is determined by shear displacement between stereocilia multiplied by Sin(Θ), where Θ is the tip link inclination angle [53]. Therefore, the extent of adaptation depends on the geometry of the MET apparatus. (B, left) If a shorter PCDH15–PCDH15 tip link is formed without changes to the stereocilia separation at the level of a tip link, Θ would decrease resulting in a smaller Δx and a larger extent of adaptation. (B, right) If stereocilia are closer to one another during link regeneration because of an increased number of shorter side links, the angle Θ may increase, decreasing the extent of adaptation. It is not possible to detect changes in Θ based on immuno-SEM images. (C) Replacement of CDH23 with PCDH15 at the upper end of the tip link may introduce to the MET apparatus an element with nonlinear stiffness (K2, red). For example, it may occur if myosin motors connecting PCDH15 to the actin core of stereocilium would not withstand tension above a certain threshold and “slide down” along the actin core. Nonlinear force–displacement relationship of the system (red graph on the right panel) would result in a decreased extent of adaptation, similar to the one observed in our experim [file pbio.1001583.s008.tif]
